# FUS Selectively Facilitates circRNAs Packing into Small Extracellular Vesicles within Hypoxia Neuron

**DOI:** 10.1002/advs.202404822

**Published:** 2024-06-26

**Authors:** Jiankun Zang, Yousheng Wu, Xuanlin Su, Kaiwei Cai, Man Ke, Niu He, Huili Zhu, Zefeng Tan, Jielin Zhu, Wensheng He, Min Peng, Shiqing Zhang, Hongcheng Mai, Anding Xu, Dan Lu

**Affiliations:** ^1^ Department of Neurology and Stroke Center The First Affiliated Hospital of Jinan University Guangzhou Guangdong 510632 China; ^2^ Department of Neurology The First People's Hospital of Foshan Foshan 528000 China; ^3^ Clinical Neuroscience Institute The First Affiliated Hospital of Jinan University Guangzhou 510632 China; ^4^ Key Lab of Guangzhou Basic and Translational Research of Pan‐vascular Diseases The First Affiliated Hospital of Jinan University Guangzhou 510632 China; ^5^ Department of Neurology The Second People's Hospital of Shunde Foshan 528300 China; ^6^ JNU‐HKUST Joint Laboratory for Neuroscience and Innovative Drug Research College of Pharmacy Jinan University Guangzhou 510632 China; ^7^ Department of Neurology Sun Yat‐Sen Memorial Hospital Sun Yat‐Sen University Guangzhou 510120 China

**Keywords:** circular RNAs (circRNAs), Fused‐in‐Sarcoma protein (FUS), Hypoxia, Small extracellular vesicles (sEVs), Stress granules

## Abstract

Small extracellular vesicles (sEVs) contain abundant circular RNAs (circRNAs) and are involved in cellular processes, particularly hypoxia. However, the process that packaging of circRNAs into neuronal sEVs under hypoxia is unclear. This study revealed the spatial mechanism of the Fused in Sarcoma protein (FUS) that facilitates the loading of functional circRNAs into sEVs in hypoxia neurons. It is found that FUS translocated from the nucleus to the cytoplasm and is more enriched in hypoxic neuronal sEVs than in normal sEVs. Cytoplasmic FUS formed aggregates with the sEVs marker protein CD63 in cytoplasmic stress granules (SGs) under hypoxic stress. Meanwhile, cytoplasmic FUS recruited of functional cytoplasmic circRNAs to SGs. Upon relief of hypoxic stress and degradation of SGs, cytoplasmic FUS is transported with those circRNAs from SGs to sEVs. Validation of FUS knockout dramatically reduced the recruitment of circRNAs from SGs and led to low circRNA loading in sEVs, which is also confirmed by the accumulation of circRNAs in the cytoplasm. Furthermore, it is showed that the FUS Zf_RanBP domain regulates the transport of circRNAs to sEVs by interacting with hypoxic circRNAs in SGs. Overall, these findings have revealed a FUS‐mediated transport mechanism of hypoxia‐related cytoplasmic circRNAs loaded into sEVs under hypoxic conditions.

## Graphical abstract

This study aimed to unravel the role of FUS in the loading of functional circRNAs into neuronal sEVs under hypoxic conditions, which involves their aggregations in SGs and specific interaction between the FUS Zf_RanBP domain to regulate circRNA sorting in sEVs.

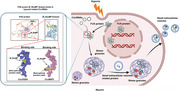

## 1 Introduction

Extracellular vesicles (EVs) are membrane‐bound compartments secreted by cells, carrying a diverse range of cargos, including RNAs, proteins, lipids, and DNAs. These EVs serve as important mediators of intercellular and inter‐organism communication, attracting significant attention due to their unique biology and roles in cell‐cell signaling.^[^ ^1^ ^]^ Small extracellular vesicles (sEVs) are particularly recognized for their ability to transport RNAs to designated recipient cells.^[^ ^2^ ^]^

Previous studies have highlighted the crucial role of RNA‐binding proteins (RBPs) in various biological processes, modulating the fate of coding and non‐coding RNAs by interacting with different stages of the RNA lifecycle.^[^ ^3^ ^]^ Several RBPs have been identified in cellular sEVs in response to stress, especially hypoxia.^[^ ^4^, ^5^ ^]^ Our recent findings hint a remarkable up‐regulation of Fused in Sarcoma protein (FUS) with a high rank in the hypoxic neuronal sEV proteomics.^[^ ^6^ ^]^ As a well‐known and highly conserved RBP, numbers studies have implicated FUS in regulating cell fate by interaction with RNAs, especially circRNAs.^[^ ^7^, ^8^, ^9^ ^]^ And circRNAs are crucial in regulating gene expression in hypoxic recipient cells.^[^ ^10^, ^11^ ^]^ Recent research has shed light on the significant enrichment of hypoxia‐related CircOGDH (circular RNA derived from oxoglutarate dehydrogenase, hsa_circ_0 003340) within sEVs, surpassing their sEVs concentration by several orders of magnitude under normoxic conditions.^[^ ^11^ ^]^ However, the precise nature of the interaction between FUS and hypoxia‐related circRNAs as well as their involvement in the transition from the cytoplasm to sEVs under hypoxic stress remain unexplored.

For another, stress granules (SGs) are unique, membraneless cellular structures within cells formed through a process of liquid‐liquid phase separation, allowing cells to adapt rapidly to the changes in stress environments^[^ ^12^ ^]^ Recent research has revealed that cytoplasmic RNAs can be recruited into SGs during stress conditions,^[^ ^13^, ^14^, ^15^ ^]^ including circRNAs.^[^ ^16^, ^17^, ^18^ ^]^ FUS is a well‐known RNA‐binding protein and plays a critical role in SGs formation.^[^ ^16^, ^19^, ^20^ ^]^ The emerging evidences have further indicated that FUS can interreact with circRNAs in the SGs of the tumor tissues core which is under hypoxia condition, and involved in tumor progression.^[^ ^16^ ^]^ However, the impact of FUS‐circRNA interaction in SGs during hypoxia on ischemic stroke is still large unknown.

By exploring the interaction of FUS with the specific circRNAs which are accumulated in SGs and subsequent transported into hypoxic sEVs (HypEV) during hypoxia and reperfusion, we uncovered a novel mechanism for the selective loading process of circRNA cargos into HypEVs. This mechanism could contribute to develop the strategies mitigating cell damage during hypoxic conditions, opening new avenues for future research and treatment.

## 2 Results

### 2.1 FUS Protein Involves in the Formation of HypEVs through the Assistance of SGs

**Figure 1:**
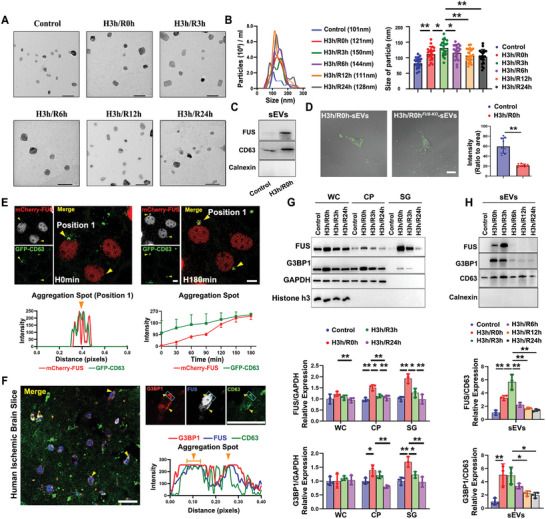
Hypoxia‐induced translocated FUS involves in the formation of HypEVs with the assistance of SGs. A) TEM images of the sEVs in each group. The sEVs are extracted by the ultrafiltration‐precipitation method. Control represents sEVs derived from control cells; H3h/R0h represents sEVs derived from cells subjected to 3 h of hypoxia without reperfusion; H3h/R3h represents sEVs derived from cells subjected to 3 h of hypoxia followed by 3 h of reperfusion; H3h/R6h represents sEVs derived from cells subjected to 3 h of hypoxia followed by 6 h of reperfusion; H3h/R12h represents sEVs derived from cells subjected to 3 h of hypoxia followed by 12 h of reperfusion; H3h/R24h represents sEVs derived from cells subjected to 3 h of hypoxia followed by 24 h of reperfusion. Scale bar, 200 nm. B) Nanoparticle tracking analysis (NTA) of the concentration and particle size measurement for these EVs purified from the six groups corresponding to Figure 1a. Median values from three independent experiments were plotted for NTA result; Particle size comparation of EVs was performed by measuring TEM images of 20 vesicles per condition from three independent experiments. *p < 0.05; **p < 0.01. C) Immunoblots show the level of FUS in sEV lysates from cells exposed to normal and hypoxic conditions. CD63 was used as the marker for sEVs; Calnexin was used as the negative marker for sEVs. D) Images showing the uptake of sEVs by primary neurons that were subjected to hypoxic conditions and subsequently incubated with H3h/R0h‐sEVs and H3h/R0h^FUS‐KO^‐sEVs for 24 h. The green spots indicate PKH67‐labeled sEVs particles. H3h/R0h‐sEVs indicates sEVs derived from the normal SH‐SY5Y cells exposed to 3 h of hypoxia without reperfusion; H3h/R0h^FUS‐KO^‐sEVs indicates sEVs derived from the FUS‐KO SH‐SY5Y cells exposed to 3 h of hypoxia without reperfusion. *N* = 9 cells from three independent experiments for measurement. **p < 0.01. Scale bar, 20 µm. E) Excerpts from a time‐lapse movie taken at 60 min intervals during the 180 min‐hypoxia process. Yellow arrows indicate long‐lasting localization of GFP‐CD63 and mCherry‐FUS in transfected cells. H0min and H180min indicate cells stressed under hypoxia for 0 and 180 min, respectively. The remaining time points images are presented in Figure S1 (Supporting Information). The fluorescence intensity quantification of the position area in H180min image (left lane) and in each time point (right lane) are shown in the bottom diagrams respectively. The overlap of different channels indicates co‐localization between molecules. Scale bar, 20 µm. F) Co‐localization staining of FUS, SGs marker protein G3BP1 and sEVs marker protein CD63 in the brain tissue sections of ischemic stroke patients with hemorrhagic transformation. Yellow arrows indicate the co‐staining points of G3BP1, FUS, and CD63. The diagrams indicate the quantification of the fluorescence intensity of each signal in the blue rectangles. The overlap of different channels indicates co‐localization between molecules. Scale bar, 200 µm. G) Immunoblots show expression levels of FUS and G3BP1 in the whole cell (WC), cytoplasm (CP) and stress granule (SG) lysates from cells subjected to hypoxia and reperfusion at different time points. Histone h3 was used as the positive control for nuclear lysates. Quantification of immunoblot from three independent experiments (*N* = 3). The WC and CP values were normalized by GAPDH; the SG values were normalized by input (WC) GAPDH. *p < 0.05; **p < 0.01. H) Immunoblots show expression levels of FUS and SGs marker protein G3BP1 in sEVs lysates from cells subjected to hypoxia and reperfusion at different time points. Calnexin was used as the negative marker for sEVs. Quantification of immunoblot from three independent experiments and values were normalized by CD63 (*N* = 3). *p < 0.05; **p < 0.01.

To determine the impact of hypoxic stress on the release of neuronal sEVs, we initially isolated sEVs from SH‐SY5Y cells exposed to different conditions, including normoxic oxygen, hypoxia for 3 h without reperfusion (H3h/R0h), and hypoxia for 3 h followed by 3, 6, 12, and 24 h of reperfusion (H3h/R3h, H3h/R6h, H3h/R12h, and H3h/R24h). The transmission electron microscopy (TEM, **Figure** **1**A) and nanoparticle tracking analysis (NTA, Figure 1B) indicated that the particle size of generated sEVs was enlarged after hypoxia, reaching the peak at 3 h reperfusion and gradually decreases. Notably, we noted a markedly higher abundance of FUS in HypEVs than that in normal sEVs (NorEVs) in Figure 1C, and a preference of recipient primary neurons for internalizing HypEVs derived from wild‐type (WT) cells rather than those from FUS knockout (FUS‐KO) cells (Figure 1D), suggesting a potential role of FUS in HypEVs generation and transport processes. To validate this possibility, the continuous live imaging of SH‐SY5Y cells which stably expressed both FUS and the sEVs marker protein CD63 was performed under hypoxic conditions. As shown in Figure 1E and Figure S1 (Supporting Information), the normal nuclear FUS protein was found to migrate out of the nucleus during hypoxia and significantly accumulated with CD63 in cytoplasm. Considering the established role of FUS as a nucleoprotein, this migration highlights its unique distribution pattern in HypEVs. However, the “aggregative spots” accumulated by FUS and CD63 seem to appear much larger than any internal intermediates of sEVs.

Given the known phase‐separation properties of FUS and its involvement in SGs formation^[^ ^,21^ ^]^ we proposed that these “aggregative spots” recruiting FUS and sEVs’ markers may be the hypoxia‐induced SGs. By using the human brain tissue sections obtained from ischemic stroke patients, we found a consistent co‐localization between FUS, CD63, and the SGs marker protein G3BP1 (Figure 1F). Subsequently, considering the formation of SGs is spatially specific, and they are unable to maintain stability within the cytoplasm after the alleviation of stress, we conducted a further investigation into the temporal and spatial correlation between FUS and SGs abundance during hypoxia and reperfusion periods. The lysis samples of distinct cell compartments, including the whole cell (WC), cytoplasm (CP), stress granule (SG), nucleus (NC), and sEVs, were harvested from the SH‐SY5Y cells exposed to hypoxia with or without reperfusion. We observed a significant elevation of FUS abundance in cytoplasm, especially in SGs samples, and a remarkable decrease of FUS in nuclear following exposure to hypoxia (Figure 1G; Figure S2, Supporting Information). However, after reperfusion and relieving stress condition, the cytoplasmic FUS was decreased as the reduction of G3BP1 within 24 h, particularly pronounced in SGs (Figure 1G). It suggests a notable translocation of FUS from nucleus to cytoplasm during hypoxia, and mainly accumulated into SGs simultaneously. Whereas, the FUS abundance in cytoplasm was highly consisted with the generation and disassemble of SGs during hypoxia and reperfusion periods.

Intriguingly, through our comparative analysis of sEV samples, we noted that contrary to the cellular compartments results, the highest level of FUS abundance in HypEVs was found in 3 h reperfusion group (Figure 1H), which coincided with the time point that substantial decrease of cytoplasmic G3BP1 and FUS (Figure 1G). Consequently, this pattern implies that the packaging of FUS into sEVs primarily occurs after the majority of SGs have undergone degradation, which highlights a potential connection between the generation of SGs and the HypEVs cargos loading process, and suggests FUS involved in sEVs formation need SGs assistance.

### 2.2 FUS Mediates the Recruitment of circRNAs into SGs During Hypoxia

**Figure 2:**
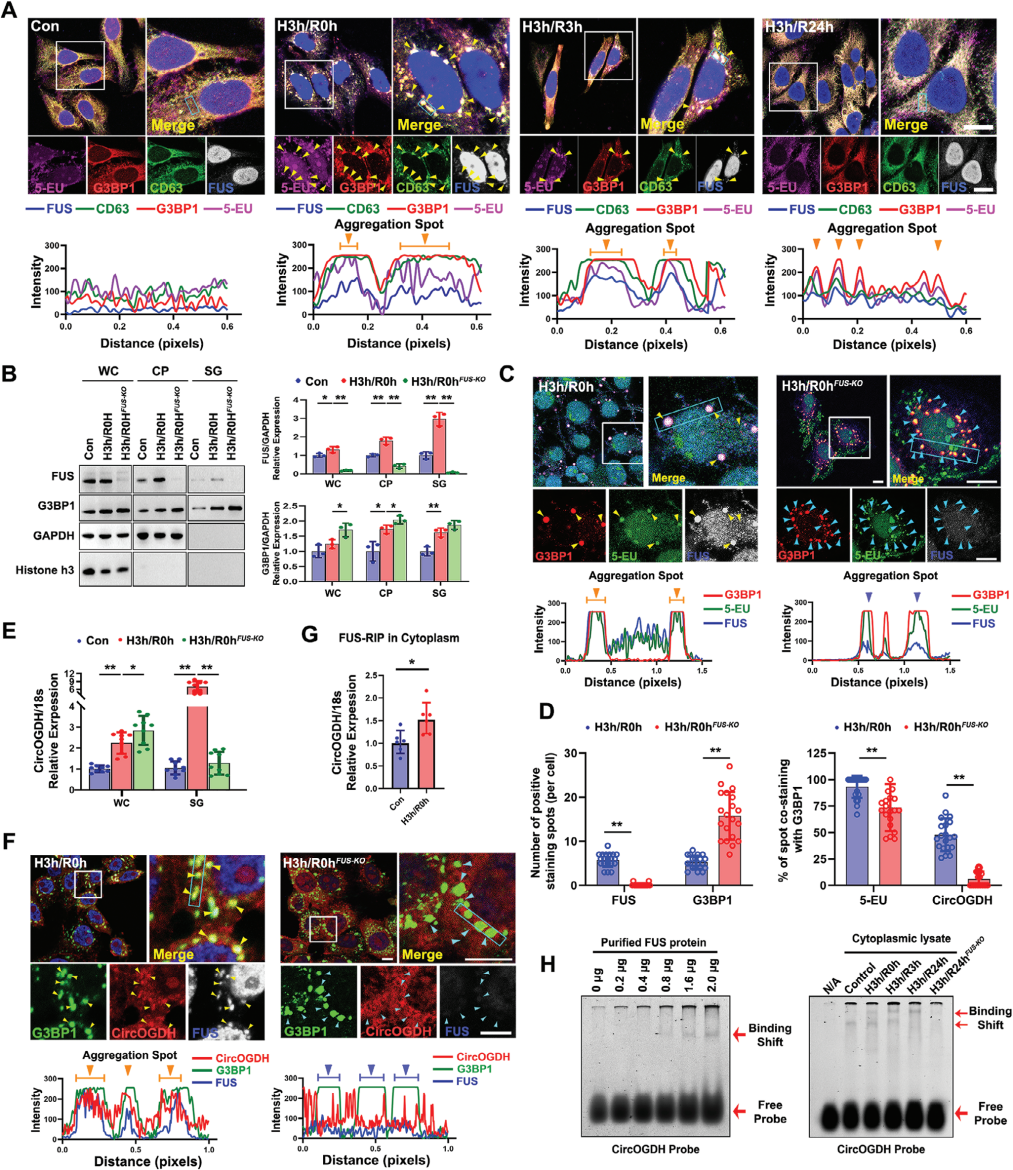
FUS mediates the recruitment of hypoxia‐related circRNAs into SGs under hypoxic conditions. A) Co‐localization staining of FUS, G3BP1, and CD63 with 5‐EU labeled nonspecific RNAs in cells subjected to hypoxia and reperfusion at different time points. Quantification of the staining spots presented in Figure S3 (Supporting Information). Yellow arrows indicate co‐staining points of G3BP1, FUS, CD63, and 5‐EU. The bottom diagrams indicate the quantification of the fluorescence intensity of each signal in the blue rectangles. The overlap of different channels indicates co‐localization between molecules. Scale bar, 20 µm. B) Immunoblots show expression levels of FUS and G3BP1 in whole cell (WC), cytoplasm (CP) and SGs lysates from Control, H3h/R0h and H3h/R0h^FUS‐KO^ cells. H3h/R0h^FUS‐KO^ represents FUS‐KO cells under 3 h of hypoxia without reperfusion. Histone h3 was used as the positive controls for nuclear lysate. Quantification of immunoblot from three independent experiments (*N* = 3). The WC and CP values were normalized by GAPDH; the SG values were normalized by input (WC) GAPDH. *p < 0.05; **p < 0.01. C) Co‐localization staining of FUS, G3BP1, with 5‐EU nonspecific RNAs in normal cells and FUS‐KO cells subjected to hypoxic condition without reperfusion. Yellow arrows indicate co‐staining points of G3BP1, FUS, and 5‐EU; blue arrows indicate co‐staining points of G3BP1 and 5‐EU without FUS. The bottom diagrams indicate the quantification of the fluorescence intensity of each signal in the blue rectangles. The overlap of different channels indicates co‐localization between molecules. Scale bar, 20 µm. D) Quantification of the number of positively stained spots for FUS and G3BP1 per cell (left lane), and the percentage of the G3BP1‐positive staining spots (right lane), corresponding to Figure 2C. *N* = 20 cells from three independent experiments for measurement. **p < 0.01. E) Relative levels of CircOGDH in whole cell (WC) and SGs samples from Control, H3h/R0h, and H3h/R0h^FUS‐KO^ cells. Quantification of CircOGDH Ct values obtained from three biological replicates with three technical replicates (*N* = 9). 18S rRNA was used to normalize Ct values. *p < 0.05; **p < 0.01. F) Co‐localization staining of FUS and G3BP1 with CircOGDH in normal cells and FUS‐KO cells subjected to hypoxic conditions without reperfusion. Yellow arrows indicate the co‐staining points of G3BP1, FUS, and CircOGDH; blue arrows indicate the co‐staining points without FUS. The bottom diagrams indicate the quantification of the fluorescence intensity of each signal in the blue rectangles. The overlap of different channels indicates co‐localization between molecules. Scale bar, 20 µm. G) Relative levels of CircOGDH in the FUS pull down sample from Control and H3h/R0h cell cytoplasmic lysates. Quantification of CircOGDH Ct values from three biological replicates with two technical replicates (*N* = 6). 18S rRNA was used to normalize Ct values. *p < 0.05. H) EMSA detection of the interaction of CircOGDH probe with purified FUS peptides at different concentrations (left lane) and cytoplasmic lysates (right lane) from different treatment cells.

As known, SGs formation is often accompanied by the recruitment of cytoplasmic RNAs during stress. If the hypoxia‐induced SGs are involved in the sEVs generation, then the process of cytoplasmic RNA recruitment into SGs may also be linked to the RNA cargos’ transport of sEVs. To delve deeper into this, we explored the intracellular localization of RNAs during hypoxia by using a comprehensive 5EU‐click chemistry method for nonspecific cytoplasmic RNA labeling. Our findings indicated that FUS, CD63 and the nonspecific RNAs labeled by 5‐EU accumulated prominently within SGs under hypoxic conditions. However, following the alleviation of hypoxia and upon 3 h of reperfusion, the colocalization of FUS, CD63, and 5‐EU within SGs reduced obviously. After 24 h of reperfusion, the prominent aggregation spots of these three were no longer visible, and instead, small and tiny co‐staining paticles were observed  (**Figure** **2**A; Figure S3, Supporting Information). Subsequently, by establishing the SH‐SY5Y^FUS‐KO^ cell line, we further observed the influence of FUS on the SGs formation and cytoplasm RNAs recruitment under hypoxic conditions. Surprisingly, we noted that instead of impairing the generation of SGs, FUS knockout resulted in a significant increase in G3BP1 expression in various cellular components during hypoxia (Figure 2B), and the immunofluorescence results also indicated that knocking out FUS further promoted SGs assembly under hypoxic condition (Figure 2C,D, left lane). Nevertheless, knocking out FUS only partially blocked the aggregation of cytoplasmic RNAs in SGs, a large amount of 5‐EU were still able to be recruited into SGs after hypoxia (Figure 2C,D, right lane).

On the other hand, we selected a circRNA, CircOGDH, which has been proven to be transported by neuronal HypEVs and is highly correlated with functional prognosis of hypoxic neural in our previous report ^[^ ^11^ ^]^ as the subsequent study subject to further explore the impact of FUS on specific RNAs during hypoxic conditions. We observed the abundance of CircOGDH and its interaction with FUS and SGs during hypoxia and reperfusion periods. As presented in Figure S4A (Supporting Information), the intracellular CircOGDH exhibited a significant increase under hypoxic conditions, followed by a gradual decline after reperfusion, especially in the SGs samples. Similarly, we observed a notable accumulation of CircOGDH along with FUS, and G3BP1 after exposure to hypoxia, which gradually dissipated as SGs disassembled within 3–6 h after reperfusion (Figure S4B, Supporting Information). However, knocking out FUS led to a significant elevation of CircOGDH in whole cell samples (increased by 1.6 fold), and triggered an obvious decline in SGs samples simultaneously (reduced by 5 fold, Figure 2E). The immunofluorescence images indicated a consistent outcome, suggesting that without FUS involvement, CircOGDH failed to be recruited into SGs and instead accumulated in the cytoplasm (Figure 2F,D). Thus, our findings demonstrate that FUS greatly contributes to the recruitment of CircOGDH toward into SGs under hypoxic condition, but CircOGDH remains in the cytoplasm after FUS knockout.

To confirm the FUS‐specific mediation of CircOGDH recruitment into SGs during hypoxia, we utilized FUS‐RIP qPCR assay to reveal an interaction between FUS and CircOGDH, which can be enhanced by hypoxia (Figure 2G). We further employed the electrophoretic mobility shift assay (EMSA) to verify the unique interaction between the purified FUS peptides and the RNA probe of CircOGDH. As shown in Figure 2H (left lane), the FUS peptides‐dependent increase in binding shift provides definitive evidence of the specific interaction between FUS and CircOGDH. Subsequently, we examined the interaction of the CircOGDH probe with cytoplasmic lysates from cells in control, H3h/R0h (3 h hypoxia with 0‐hour reperfusion), H3h/R3h, H3h/R24h, and H3h/R24h^FUS‐KO^ groups by EMSA, and found two distinct binding bands formed by CircOGDH probe and cell lysates. Noteworthy, the binding bands showed an upward migration tendency as reperfusion time increased, and the specific bands were significantly diminished in FUS‐KO cell lysates (Figure 2H, right lane). These results mention that FUS potentially involved in the selective transport of sEV circRNAs by facilitating the recruitment of those circRNAs into SGs during hypoxia.

**Figure 3:**
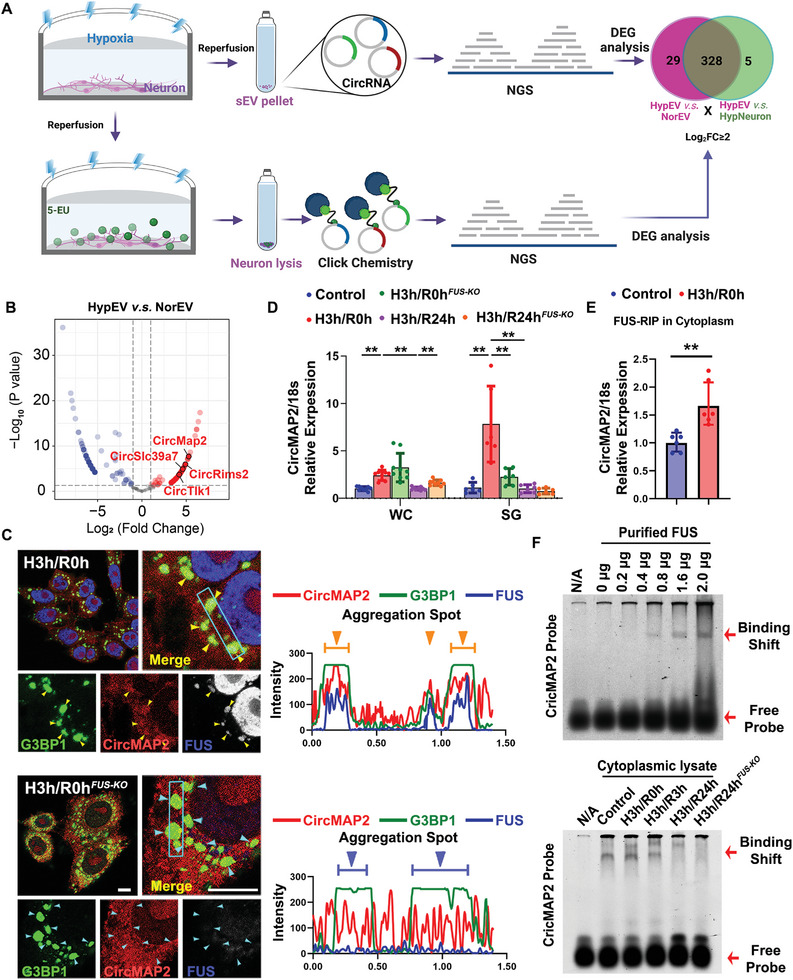
FUS may involve in the recruitment of a class of sEV‐transported circRNAs into hypoxia‐induced SGs. A) Schematic representation of RICK‐RNA‐seq analysis of newborn RNAs generated in primary neurons subjected to hypoxia and reperfusion, and total‐RNA‐seq analysis of sEVs derived from primary neurons exposed to hypoxia and reperfusion. The Venn diagram on the right shows the overlapped differentially expressed circRNAs between HypEVs and hypoxic neurons (HypEV *v.s*. HypNeuron), and between HypEV and NorEV. Differentially expressed circRNAs are defined by log_2_FC >2. B) Volcano plots illustrating differentially expressed circRNAs between theHypEVs and NorEVs. Four hub‐function‐related differentially expressed circRNAs are marked in the diagram, including CircMAP2, CircRims2, CircTlk1, and CircSlc39a7. Functional enrichment analysis of the differential expressed circRNAs was presented in Figure S5 (Supporting Information). C) Co‐localization staining of FUS and G3BP1 with CircMAP2 in normal cells and FUS‐KO cells subjected to hypoxic conditions without reperfusion. Yellow arrows indicate the co‐staining points of G3BP1, FUS, and CircMAP2; blue arrows indicate the co‐staining points without FUS. The right diagrams indicate the quantification of the fluorescence intensity of each signal in the blue rectangles. The overlap of different channels indicates co‐localization between molecules. Scale bar, 20 µm. D) Relative levels of CircMAP2 in whole cell (WC) and SGs samples from Control, H3h/R0h, and H3h/R0h^FUS‐KO^ cells. Quantification of CircMAP2 Ct values obtained from three biological replicates with three technical replicates in WC samples (*N* = 9); three biological replicates with two technical replicates in SG samples (*N* = 6). 18S rRNA was used to normalize Ct values. **p < 0.01. E) Relative levels of CircMAP2 in the FUS pull‐down sample from Control and H3h/R0h cell cytoplasmic lysates. Quantification of CircMAP2 Ct values from three biological replicates with two technical replicates (*N* = 6). 18S rRNA was used to normalize Ct values. *p < 0.05. F) EMSA detection of CircMAP2 probe with purified FUS peptides (top lane) and cytoplasmic lysates from different treated cells (bottom lane).

To demonstrate that the FUS‐mediated recruitment of circRNAs into SGs is not limited to CircOGDH, we employed capture of the newly transcribed RNA interactome using click chemistry (RICK)^[^ ^22^ ^]^ to identify nascent functional circRNAs that were transported by the HypEVs (**Figure** **3**A, left lane). In this section, all the HypEVs used for RICK‐seq were derived from H3h/R3h neurons, which have a highest abundance of FUS and SGs components. By comparing the differentially expressed nascent circRNAs in hypoxic cells and HypEVs samples and those in HypEVs and NorsEVs samples, we identified that 328 out of 367 differentially expressed nascent circRNAs between NorEVs and HypEVs, and which were also present in differential circRNA expression profile between Hypoxic cells and HypEVs (Figure 3A, right lane). In addition, functional enrichment analysis of the overlapped differentially expressed nascent circRNAs in HypEVs revealed that the parent genes of these circRNAs were predominantly involved in neuronal function‐related biological processes, including intercellular communication, neuronal morphology, and the maintenance of axonal function (Figure S5, Supporting Information). Therefore, we deduced that the majority of differentially expressed circRNAs in HypEVs are selectively transported from cells during hypoxia and reperfusion phases, as opposed to NorEVs. Most of these circRNAs are highly implicated in the regulation of neuronal functions.

Four representative circRNAs, including CircMAP2, CircRims2, CircSlc39a7, and CircTlk1, were identified in HypEVs. One of the top significantly enriched circRNA, CircMAP2, was selected for further validation (Figure 3B). Similar to CircOGDH, the hypoxia‐related CircMAP2 also exhibited a specific interaction with FUS during the hypoxia and reperfusion periods. The intracellular CircMAP2 exhibited a significant increase under hypoxic condition, followed by a gradual decline after reperfusion, especially in the SGs samples (Figure S6A, Supporting Information). And the co‐localization detection further indicated that CircMAP2 primarily aggregated in SGs along with FUS under hypoxic condition, and gradually dissipated as SGs disassembled within 3–6 h after reperfusion (Figure S6B, Supporting Information). However, knocking out FUS led to an increased generation of SGs during hypoxia and halted the accumulation of CircMAP2 within SGs (Figure 3C). RT‐qPCR results also suggested that blocking FUS induced an elevation of CircMAP2 in the whole cell samples, but it was no longer recruited into SGs (Figure 3D). Additionally, FUS‐RIP qPCR results suggested an enhanced interaction between FUS and the CircMAP2 in hypoxic cells (Figure 3E). The EMSA assay also revealed a specific interaction between the CircMAP2 probe and purified FUS peptides (Figure 3F, top lane). This interaction was further evidenced by the observation of two upward migrating bands in cell lysates from hypoxia and reperfusion conditions. However, when FUS was knocked out, the formation of these binding bands was significantly diminished (Figure 3F, bottom lane).

Thus, the impact of FUS on the recruitment of nonspecific RNAs into SGs might be limited, while it is essential for the aggregation of a class of functional circRNAs into SGs during hypoxia and reperfusion conditions. These findings imply that FUS might further mediate a selective transport mechanism for circRNA cargos within HypEVs.

### 2.3 FUS Mediates the Specific Delivery of circRNAs into HypEVs via SGs Translocation

**Figure 4:**
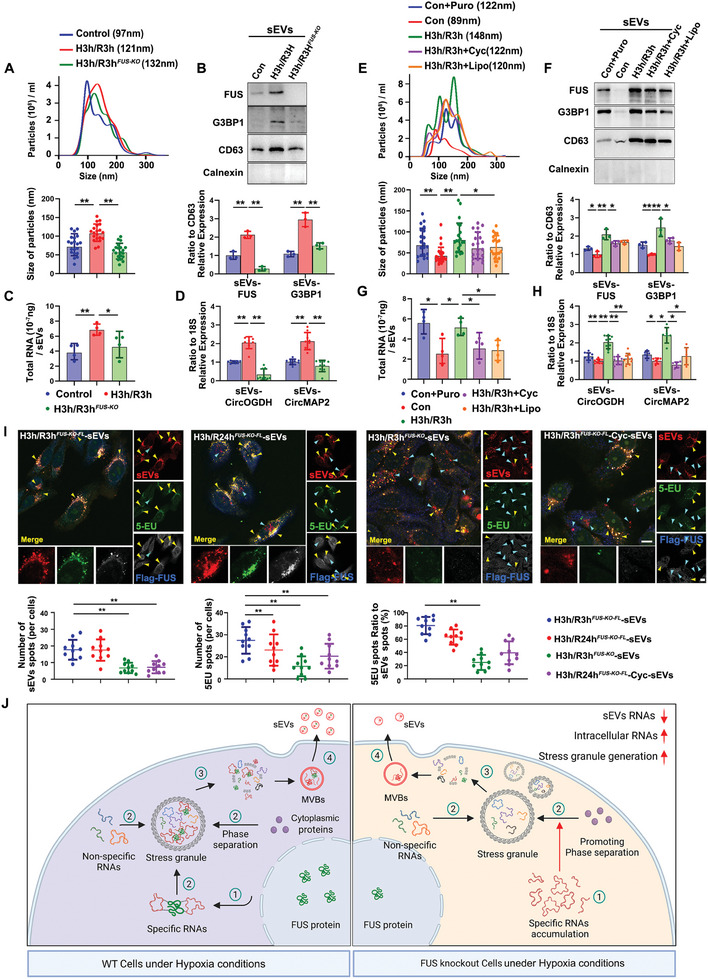
FUS and SGs collaboratively involve in the regulatory process of loading hypoxia‐related circRNA cargos into HypEV. A) NTA of the concentration and particle size measurement for these EVs purified from Control, H3h/R3h, and H3h/R3h^FUS‐KO^ cells (top lane). Median values from three independent experiments were plotted. Graphs show particle size of those EVs calculated from a total of 20 vesicles per condition from three independent experiments (*N* = 20, bottom lane). **p < 0.01. B) Immunoblots show expression levels of FUS and SGs marker protein G3BP1 in sEVs lysates from different sources of cells, corresponding to Figure 3C. Quantification of immunoblot from three independent experiments and values were normalized by CD63 (*N* = 3). **p < 0.01. C,D) Total RNA concentration of each sEVs isolated from different treatment cells (C), and relative levels of CircOGDH and CircMAP2 in those sEVs (D), corresponding to Figure 4A. The total RNA quantity measured by NanoDrop from four independent experiments (*N* = 4). Quantification of CircOGDH and CircMAP2 Ct values were normalized by respective 18S values from three biological replicates with three technical replicates (*N* = 9). *p < 0.05; **p < 0.01. E) NTA traces and particle size measurement for these EVs purified from normal and hypoxic cells with or without SGs modulator treatment. Con+Puro indicates the cells treated with 30 µg mL^−1^ puromycin for 3 h without stressing; H3h/R0h+Lipo and H3h/R0h+Cyc refer to the cells treated with 30 µm lipoamide or 200 µg mL^−1^ cycloheximide during 3 h of hypoxia without reperfusion. Median values from three independent experiments were plotted for NTA result (top lane); Particle size comparation of EVs was performed by measuring 20 vesicles per condition from three independent experiments (bottom lane). *p < 0.05; **p < 0.01. F) Immunoblots show expression levels of FUS and SGs marker protein G3BP1 in sEVs lysates from different treatment cells, corresponding to Figure S9A (Supporting Information). Quantification of immunoblot from three independent experiments and values were normalized by CD63 (*N* = 3). **p < 0.01. G,H) Total RNA concentration of each sEVs harvested from different treatment cells (G), and relative levels of CircOGDH and CircMAP2 in those sEVs (H), corresponding to Figure 4E. The total RNA quantity measured by NanoDrop from four independent experiments (*N* = 4). Quantification of Ct values were normalized by respective 18S values from three biological replicates with three technical replicates in CircOGDH (*N* = 9) and two technical replicates in CircMAP2 (*N* = 6). *p < 0.05; **p < 0.01. I) Co‐localization staining of 5EU‐RNAs and FLAG‐FUS transported by sEVs from different sources in recipient cells. The workflow diagram is presented in Figure S9 (Supporting Information). Yellow arrows indicate co‐staining of FLAG‐FUS, 5EU‐RNAs, and PKH26‐sEVs; blue arrows indicate staining points without FLAG‐FUS. Quantification of the number of sEVs (left lane) and 5‐EU staining spots per cell (middle lane), and the percentage of 5‐EU‐positive sEVs staining spots (right lane). *N* = 10 cells from three independent experiments for measurement. **p < 0.01. Scale bar, 20 µm. J) Schematic diagram of the FUS‐mediated selective transport of specific circRNAs into sEVs via SGs during hypoxia and reperfusion periods.

To validate the hypothesis that FUS‐mediated specific recruitment of circRNA into SGs is related to the transport of circRNA cargos within HypEVs, we further examined the effects of FUS and SGs on the characterization of HypEVs and the transport of their cargos in this section. By isolating sEVs from normal, H3h/3 h, and H3h/3H^FUS‐KO^ cells, we conducted a comparison of NTA assay to assess the influence of FUS on the morphology of EVs. As shown in **Figure** **4**A, we observed a significant reduction in the particle size of H3h/3 h sEVs when FUS was knock out. Alongside the knockout of FUS, a noteworthy decrease in G3BP1 abundance was observed in sEVs from FUS‐KO cells (Figure 4B). Importantly, in comparison to the H3h/3 h group, both the total RNA cargos (Figure 4C) and hypoxia‐related circRNAs (Figure 4D) within each sEV were significantly diminished after FUS inhibition, and the alteration in those specific circRNAs was much more remarkable than in total RNAs.

We further explored the effects of SGs on the characterization of HypEVs and the transportation of their cargo. After treatment with SGs inducer (puromycin) and SGs inhibitors (cycloheximide and lipoamide) to regulate the generation of SGs, we found that promoting SGs formation in normal cells or inhibiting their formation in H3h/R3h cells led to significant changes in the particle sizes of sEVs (Figure 4E). Simultaneously, the abundance of FUS (Figure 4F), total RNAs (Figure 4G) and hypoxia‐related circRNAs (Figure 4H) within sEVs also altered. Thus, these findings demonstrate that FUS, in conjunction with SGs, participates in the generation of HypEVs and the selective transport of the RNA cargos. However, compared to SGs, FUS has an even more pronounced role in regulating the transport of those specific circRNAs within HypEVs.

To visually confirm the essential roles of FUS and stress granules in the transportation process of RNA cargos within HypEVs, we further examined their effects on sEVs‐RNA transport in recipient cells (the flow chart presented in Figure S7, Supporting Information). First, we supplemented the FLAG‐tagged FUS in FUS knockout cells to distinguish the sEVs‐transported FUS in recipient cells. We found that the RNA transport capacity of HypEVs derived from 24 h reperfusion was comparable to that observed at 3 h (Figure 4I), despite the 3 h reperfusion HypEVs have higher abundances of the FUS and G3BP1 than that in 24 h reperfusion HypEVs (Figure 1G). We believe this might be related to the gradually increasing ability of FUS to bind circRNA after reperfusion (Figures 2H and 3F), resulting in FUS effectively interacting with the hypoxia‐related RNAs during SGs degradation and maintaining the transport of these RNAs in sEVs post‐reperfusion. Additionally, inhibiting SGs production or eliminating FUS (without Flag‐FUS supplement) in donor cells considerably reduced the number of nonspecific sEVs RNAs (Figure 4I), which further demonstrated the FUS and SGs are indispensable for the sEVs RNAs cargos loading. It should be noted that blocking SG generation also led to a significant decrease in sEVs‐FUS transport. Meanwhile, we can also observe that the effect of FUS on sEVs RNA cargos transportation is more significant compared to the role of stress granules (Figure 4I). Therefore, from these observations, we concluded that the transportation of specific HypEVs circRNA cargos is facilitated by the selective recruitment of FUS and the transitional delivery through SGs. Without the assistance of FUS, the hypoxia‐related specific circRNAs cannot be recruited into SGs and accumulate in cytoplasm, causing a subsequent decrease in the amount of circRNAs in sEVs.

### 2.4 Zf_RanBP Domain is Essential for FUS‐Dependent Selective Delivery of the Hypoxia‐Related circRNA Cargos into HypEVs

**Figure 5:**
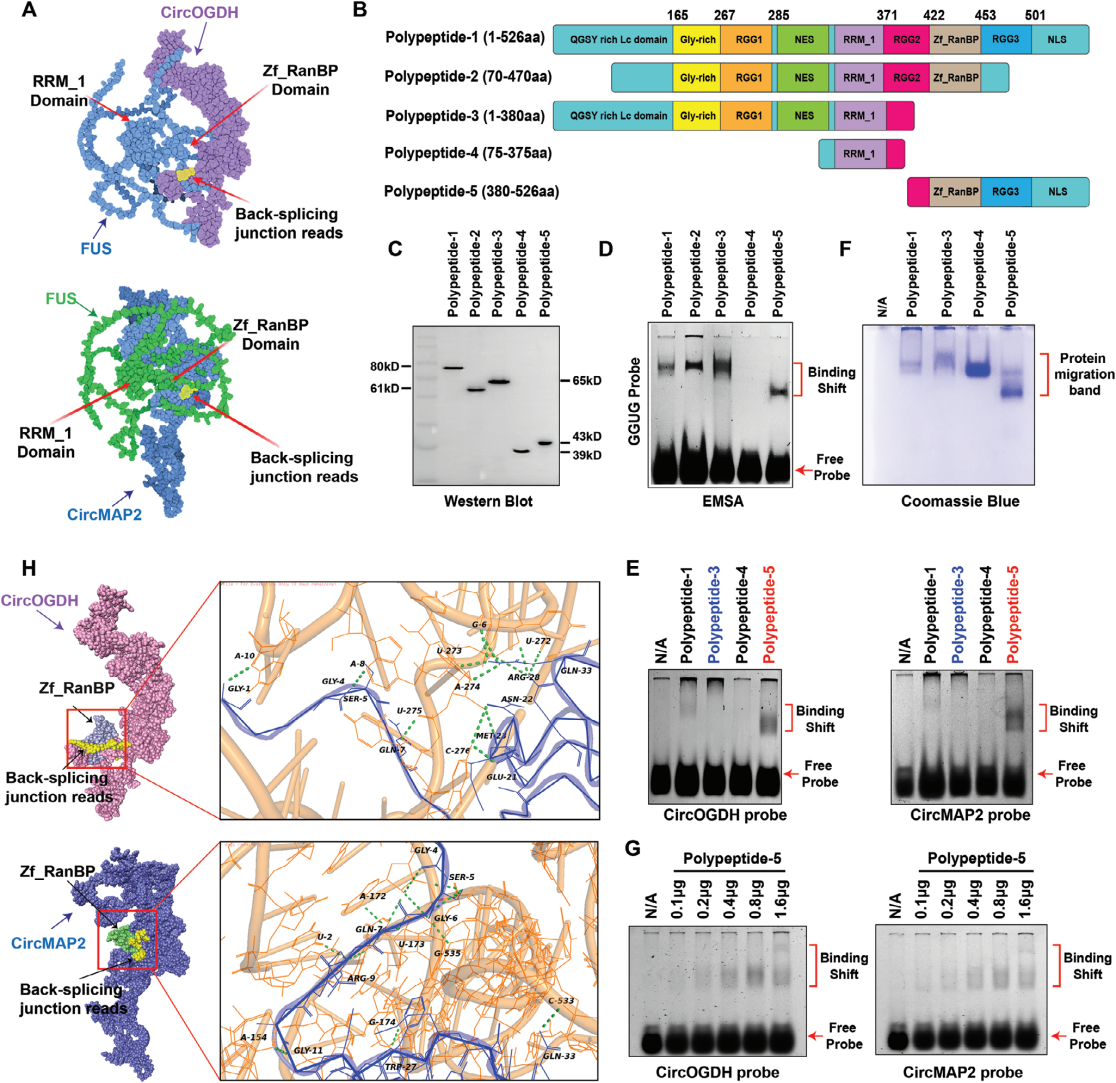
FUS selectively interacts with hypoxia‐related circRNAs via its Zf_RanBP domain, binding to the junction reads of these circRNAs. A) Schematic diagram depicting the simulated spatial structure of FUS (purple in top lane and green in bottom lane) combined with CircOGDH (top lane, blue) and CircMAP2 (bottom lane, blue). The junction reads of circRNAs are marked in yellow. B) Schematic representation of the construction strategy for FUS truncated peptides. C) Immunoblots verification of the five truncated FUS peptide. D) EMSA detection of five purified truncated FUS peptides interacting with FUS‐specific GGUG probe in vitro. E) EMSA detection of four purified truncated FUS peptides (except for Polypeptide‐2) interacting with the junction read‐specific probes of CircOGDH and CircMAP2 in vitro. F) Coomassie staining image of the gel after EMSA detection in Figure 5E. G) EMSA detection of the junction read‐specific probes of CircOGDH and CircMAP2 interacted with the gradient concentrations of Polypeptide‐5 in vitro. H) Schematic diagram illustrating the complex structures of the Zf_RanBP domain (blue in top lane and green in bottom lane) combined with CircOGDH (purple in top lane) and CircMAP2 (purple in bottom lane) in the left. The right lane is the high‐resolution view of the interaction regions between Zf_RanBP (blue) and junction reads of circRNAs (orange). Green dotted lines represent the covalent hydrogen bond formed between the two molecules.

To investigate how FUS recognizes and selectively binds to hypoxia‐related circRNAs, we used RNAfold and 3dRNA tools to predict and construct the spatial structures of CircOGDH and CircMAP2, and obtained the FUS structure from the AlphaFold Protein Structure Database. By ZDOCK analysis, we developed interaction complexes between both circRNAs and FUS, and observed consistent spatial proximity between Zf_RanBP domains of FUS and the junction reads of both circRNAs (**Figure** **5**A). To further dissect the functional regions of FUS involved in this interaction, we constructed five truncated FUS polypeptides, each containing different functional domains (Figure 5B), and subjected them to Western blot identification (Figure 5C). We first synthesized the probes derived from the junction reads of CircOGDH and CircMAP2, and a FUS protein‐specific recognition probe “GGUG” (Figure S8, Supporting Information), for the detection of EMSA. As presented in Figure 5D, polypeptide‐4, containing only the RRM_1 domain, failed to bind to both the GGUG probe. Since polypeptide‐2 has the functional domains same as polypeptide‐1, they have a consistent ability to interact with GGUG probes. The polypeptide‐1, 3,4, and 5 were used for the following detections. Notably, polypeptide‐3, which interacted with the GGUG probe but lacked the Zf_RanBP domain, also failed to engage with the junction probes of the hypoxia‐related circRNAs (Figure 5E,F). In contrast, polypeptide‐5, containing the Zf_RanBP domain, was specifically bound to both circRNA probes (Figure 5E‐G), suggesting the polypeptide‐5 possesses a distinctive affinity for the junction reads of the hypoxia‐related circRNAs. Next, molecular docking of the Zf_RanBP domain with CircOGDH and CircMAP2 revealed specific binding to the junction reads of circRNAs (Figure 5H). We then examined the interaction between FUS and three other circRNAs (CircRims2, CircSlc39a7, and CircTlk1), and found that the Zf_RanBP domain could specifically bind to their junction reads (Figure S9, Supporting Information). These data confirm the crucial role of Zf_RanBP in FUS's interaction with circRNAs through specific recognition of the junction reads of circRNAs.

**Figure 6:**
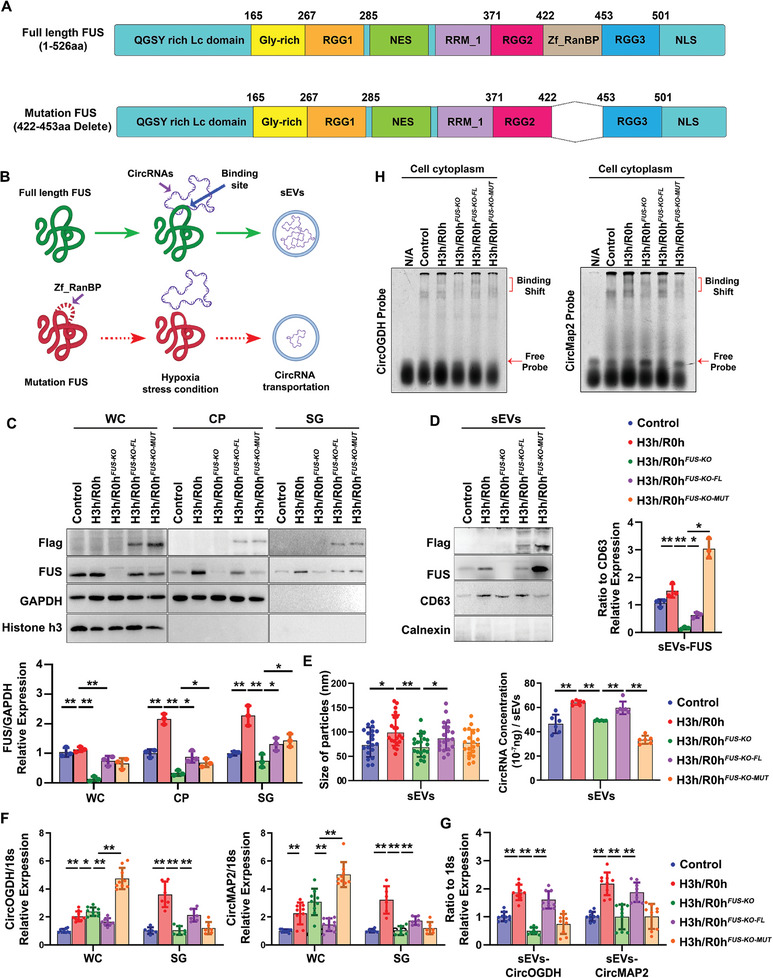
Zf_RanBP domain is essential for FUS facilitating the hypoxia‐related circRNAs transport into HypEVs. A) Schematic representation of the FLAG‐tagged full‐length FUS (FL) and Zf_RanBP deleted mutant FUS (MUT) plasmids. Those plasmids were transfected into FUS‐KO cells to generate FUS‐KO‐FL cells and FUS‐KO‐MUT cells. B) Schematic illustrating the impact of the Zf_RanBP domain of FUS on the transport of specific circRNAs into HypEVs. C) Immunoblots verification of the exogenous FUS in different cellular components of the FUS‐KO‐FL and FUS‐KO‐MUT cells exposed to 3 h hypoxia without reperfusion. Quantification of immunoblot from three independent experiments and values were normalized by GAPDH (*N* = 3). *p < 0.05; **p < 0.01. D) Immunoblots verification of the exogenous FUS in sEVs derived from FUS‐KO‐FL and FUS‐KO‐MUT cells exposed to 3 h hypoxia and 3 h reperfusion. Quantification of immunoblot from three independent experiments and values were normalized by CD63 (*N* = 3). *p < 0.05; **p < 0.01. E) Particle size analysis (left lane) and total RNA concentration (right lane) of different resources of sEVs, corresponding to Figure 6D. Particle size was calculated from a total of 20 vesicles each group from three independent experiments (*N* = 20); total RNA quantity was measured by NanoDrop from four independent experiments (*N* = 4). *p < 0.05; **p < 0.01. F) Relative levels of CircOGDH and CircMAP2 in different cellular components from the corresponding cells in Figure 6C. Quantification of CircOGDH and CircMAP2 were normalized by respective 18S from three biological replicates with three technical replicates in whole cell samples (*N* = 9) and two technical replicates in SGs samples (*N* = 6). *p < 0.05; **p < 0.01. G) Relative levels of CircOGDH and CircMAP2 in sEVs from the corresponding groups in Figure 6D. Quantification of CircOGDH and CricMAP2 were normalized by respective 18S from three biological replicates with three technical replicates (*N* = 9). *p < 0.05; **p < 0.01.

To delve deeper into the role of Zf_RanBP in FUS‐mediated cytoplasmic transport of circRNAs, we transfected full‐length FUS (FL) and Zf_RanBP‐deleted FUS (MUT) plasmids into the FUS‐KO cell lines, respectively (**Figure** **6**A,B). After stressing under hypoxic condition, we extracted lysates from different cellular components, including whole cell, cytoplasm, SGs and sEVs, and then verified the expression of FUS proteins in each sample (Figure 6C,D). The results indicated that both the exogenous FUS and the tagged protein could be detected in all components of cells transfected with FUS‐FL and FUS‐MUT plasmids, confirming the successful establishment of those two cell lines. Importantly, when comparing the characterization and abundance of RNA cargos of different sources HypEVs, we noted that the sEVs generated from the FUS‐FL transfected cells supplementary group have a significant increase in both particle size and circRNA abundance compared to the FUS‐KO stable cell line (Figure 6E). However, there were no significant differences observed in the FUS‐MUT transfection group, even though the FUS‐MUT protein was detected in both cells and small extracellular vesicles (Figure 6E). By testing the abundance of CircOGDH and CircMAP2 in different cellular components, we found that compared to the FUS‐KO stable cell lines, these two specific circRNAs were significantly reduced in the cytoplasmic samples, but highly increased in SGs and sEVs samples of cells co‐transfected with FUS‐FL plasmids (Figure 6F,G), indicating that the exogenous FUS can restore the transportation of hypoxia‐related circRNA cargos into HypEVs with the assistance of SGs. Nevertheless, the abundance of those specific circRNAs in the FUS‐MUT group have no significant recovery neither in sEVs nor in SGs, but further accumulated in the cytoplasm (Figure 6F,G). The EMSA detection further provides additional evidence that the junction probes designed for CircOGDH and CircMAP2 generated distinct binding shifts in the cell lysates in FUS‐FL supplementation group, but absence in FUS‐MUT transfected cells. This strongly suggests that the Zf_RanBP domain is essential for FUS to recognize and bind the hypoxia‐related circRNAs (Figure 6H).

In order to further explore the selective circRNA binding strategy of the Zf_RanBP domain in FUS, we employed an enhanced cross‐linking and immunoprecipitation sequencing (eCLIP‐seq) to screen the specific RNA motifs in circRNA that recognized by the Zf_RanBP domain. First, we compared the number of RNAs motif recognized by FUS in WT cells, FUS knockout cells and FUS mutation cells, respectively (Figure S10A, Supporting Information). Setting the motif length from 4 to 7 nucleotides,^[^ ^23^ ^]^ and we found 71 FUS‐related RNA motifs were detected in WT cells. Meanwhile, 149 and 109 motifs were detected in KO and MUT cells respectively, but the fold enrichment of these motifs were less than 1.1 (target % related to background %) with no preferential distribution. On the other hand, 55 unique motifs were identified in WT cells, and 23 motifs have statistically significant (*p* < 0.05) with high explicitness (at least 2/3 positions of the motif display a nucleotide with ≥ 60% probability) were selected.^[^ ^23^, ^24^ ^]^ As shown in Figure S10B (Supporting Information), the most enrichment motif is CGCAUA, and the motif CGAA can be found in CircOGDH and CircMAP2 sequences.

In summary, based on the above observations, we conclude that the Zf_RanBP domain of FUS can specifically recognize and bind to the junction reads of a subset of hypoxia‐related circRNAs. Depending on this feature, FUS can aggregates with these functional circRNAs into SGs under hypoxic conditions. Upon stress relief, these complexes are further transported into sEVs as SGs disassemble. This strategy consequently facilitates a unique hypoxia‐triggered sorting mechanism for the transportation of circRNAs within HypEVs.

## 3 Discussion

The biological effects of sEVs are inherently tied to their contents,^[^ ^1^, ^25^ ^]^ and certain RNA species, notably circRNAs, are selectively incorporated into them.^[^ ^11^, ^26^ ^]^ Recent studies have highlighted the potential of using RBPs to regulate the loading of circRNAs into sEVs, thereby influencing cellular pathological conditions.^[^ ^27^, ^28^, ^29^, ^30^ ^]^ It is worth noting that there are more than 500 RBPs reported in mammalian cells, constituting ≈25% of the protein content of sEVs.^[^ ^31^, ^32^ ^]^ RBPs exhibit selectivity for specific RNA sequence^[^ ^33^, ^34^, ^35^, ^36^ ^]^ and likely contribute to the selective transport of RNAs by sEVs.^[^ ^37^ ^]^ However, little is known about the distribution of these RBPs in different types of sEVs and their role in RNA loading. Herein, our study reveals the following key findings. First, FUS is highly abundant in HypEVs and undergoes a sorting process of RNA into sEVs with the involvement of SGs acting as intermediaries. Second, FUS interacts with formatted SGs, which in turn recruit RNAs, particularly hypoxia‐responsive circRNAs, such as CircOGDH and CircMAP2. Third, the Zf_RanBP domain of FUS plays a crucial role in its interaction with hypoxia‐related specific circRNAs, facilitating their recruitment to SGs and subsequent transport into HypEVs.

In fact, it has been reported that cellular FUS is involved in the generation, localization, and expression of circRNA, as well as in the formation of SG. For examples, FUS can promote the process of back‐splicing of circRNAs and facilitate breast cancer liver metastasis by promoting epithelial mesenchymal transition via the circRNAs/KLF5/FUS feedback loop.^[^ ^8^, ^38^ ^]^ Meanwhile, hypoxia‐induced FUS–circTBC1D14‐associated SG was involved in the progression of breast cancer.^[^ ^16^ ^]^ Besides in the cancer field, as circRNAs have been increasingly recognized for their pivotal role in the nervous system,^[^ ^39^, ^40^ ^]^ particularly in axons and dendrites of neurons.^[^ ^41^, ^42^ ^]^ Our previous study^[^ ^6^ ^]^ suggested that the FUS protein potentially regulates mitochondrial reprogramming in receptor neurons by mediating the uptake of the hypoxia‐related RNAs, CircOGDH.^[^ ^11^ ^]^ In this study, our finding that FUS mediates the transport of functional circRNAs into HypEVs uncovers a novel mechanism for regulating sEVs‐circRNA transport under hypoxic conditions in the nervous system. This finding significantly impacts our understanding of neuronal function and prognosis during acute pathological states, especially hypoxia.

SGs are membraneless organelles formed by the accumulation of liquid‐liquid phase separation proteins, including FUS, under stress condition.^[^ ^16^, ^17^, ^18^ ^]^ Initially, SGs were believed to mainly recruit mRNAs and protect them from stress‐induced damage.^[^ ^15^, ^43^, ^44^ ^]^ However, recent studies have shown that several long non‐coding RNAs (lncRNAs) and circRNAs are also specifically localized in SGs,^[^ ^14^, ^15^ ^]^ and circRNAs with higher SG‐related RBP binding ability are more likely to be enriched in SGs.^[^ ^14^ ^]^ Thus, the recruitment of specific cytoplasmic RNAs into SGs appears to be precisely regulated by SG‐associated proteins. SGs can be removed through two distinct mechanisms: autophagy‐dependent degradation and non‐degradative disassembly. Autophagy‐dependent degradation leads to the permanent loss of SGs constituent proteins and RNAs^[^ ^45^, ^46^ ^]^ while, non‐degradative disassembly involves the reversal process of liquid‐liquid phase separation, resulting in the dissociation and recycling of individual proteins and RNAs.^[^ ^47^, ^48^, ^49^, ^50^ ^]^ Interestingly, both SGs degradation processes share commonalities with sEVs generation. For instance, autophagosomes produced by SGs degradation have been implicated in the generation of MVBs, which are intracellular precursors of sEVs.^[^ ^51^, ^52^ ^]^ Ubiquitination modification promotes autophagy‐independent disassembly of SGs^[^ ^46^, ^48^ ^]^ and is also involved in regulating the sEVs secretion.^[^ ^53^ ^]^ Previous studies have suggested that SGs potentially affect the composition of RNA cargos in sEVs via autophagy‐mediated degradation.^[^ ^54^ ^]^ Although the cytoplasmic processes are yet fully understood, it is reasonable to assume that the recruitment of cytoplasmic RNAs by SGs may make a great contribution to the transport of specific sEVs‐RNA cargos during stress conditions. In our study, we proposed that FUS is highly involved in the SG‐dependent sEV transport of specific circRNAs that interact with it. Furthermore, RBP domains have specific regions that recognize junction sites in circRNAs during the splicing of linear RNAs.^[^ ^55^ ^]^ Previously, it was unclear which of the two primary functional domains of FUS, RRM_1 or Zf_RanBP, is responsible for binding to circRNAs.^[^ ^56^ ^]^ In this study, we showed that the Zf_RanBP domain, rather than the RRM_1 domain, is the primary region of FUS that recognizes specific circRNAs, consistent with earlier reports highlighting the vital role of the Zf_RanBP domain in RNA recognition.^[^ ^57^ ^]^ We demonstrated that the Zf_RanBP could specifically recognize and bind to the junction sites of certain circRNAs derived from HypEVs. This understanding adds a new regulatory target dimension to the involvement of Zf_RanBP domain in transporting these target circRNAs to sEVs.

However, it should be noted that while hypoxia is a very common stress condition associated with multiple pathological conditions, it remains to be explored whether other stressors can also induce significant SGs generation in cells and share the same pathway involving FUS and SGs in regulating circRNA loading to sEVs. Besides, further exploration in different cell lines is required to validate these findings. Overall, this novel understanding of the mechanism provides the potential to accurately assess and load the biological content of sEVs under stress conditions.

The study unveils a novel mechanism for the transport of hypoxia‐related circRNAs to sEVs during hypoxic stress, which involves their aggregation in SGs and specific interaction with the FUS Zf_RanBP domain. These findings shed light on the mechanisms underlying sEV‐mediated circRNA transport and offer insights into cellular responses to hypoxia.

## 4 Conclusion

This study aimed to unravel the role of FUS in loading circRNAs into neuronal HypEVs. By exploring the function of FUS accumulation in SGs and its interaction under stress conditions to regulate circRNA sorting in sEVs, we unveiled a novel mechanism for the transport of the hypoxia‐related specific circRNAs into HypEVs, involving their aggregation in SGs and specific interaction with the FUS Zf_RanBP domain. These findings illuminate the mechanisms underlying sEV‐mediated circRNA transport and offer insights into cellular responses to hypoxia.

## 5 Experimental Section

### Human Brain Tissue Samples

Ethical approval for this study was obtained from the Medical Ethics Committee of the First Affiliated Hospital of Jinan University. Samples of brain tissue were obtained from patients with hemorrhagic cerebral infarction who died between 1 and 3 days after stroke onset or brain tissue had to be removed for the surgery assessed by neurosurgeons (*N* = 3). The hemorrhagic transformation diagnosis was verified by pre‐operative MR scans and post‐operative pathological analysis. The samples were harvested from specific brain regions within the vascular territory of the middle cerebral artery.

### Cell Culture

The SH‐SY5Y cell lines were cultured in Dulbecco's modified Eagle's medium (DMEM) supplemented with 10% fetal bovine serum (FBS), 1% penicillin/streptomycin at a temperature of 37 °C with 5% CO_2_ in an incubator and were maintained according to standard procedures. Near confluence, cells were passaged using 0.25% trypsin with 0.02% EDTA.

For Primary neurons culture, the cells culture plates were pre‐coated with 0.1 mg ml^−1^ of Poly‐L‐Lysine for 16 h before using. The extraction steps were performed as previously study,^[^ ^11^ ^]^ and cells were derived from the cerebral cortex of Balb/c mouse embryos (E18 – E19). Briefly, the embryonic cortex was isolated and dissociated by mincing with tips followed by trypsin digestion for 15 min at 37 °C. During digestion, the tissue was treated with 1 units ml^−1^ DNase I to prevent neuronal clumping. After termination with FBS, the neurons were filtered through a 70 µm cell strainer and collected by centrifugation at 200 g for 5 min. Then, the extracted cells were plated into the pre‐coated plates at a density of 8 × 107 cells per well with DMEM/F12, 10% FBS and penicillin‐streptomycin for 4 h. After 4 h incubation, the cultures medium was replaced by the Neurobasal complete medium containing 1% penicillin/streptomycin and 2% B27 supplement. Half of the medium was replaced every 3 days with fresh Neurobasal complete medium. The primary neurons were used for following detection after 6 days culture. The pregnant mice were purchased from the Guangdong Medical Laboratory Animal Center and all procedures were conducted in accordance with the guidelines laid out in the NIH Guide (NIH Publications No. 8023, revised 1978) for the Care and Use of Laboratory Animals. Furthermore, all animal procedures were approved by the Institutional Animal Care and Use Committee of Jinan University (approval ID: 20201028‐03).

### Plasmids Extraction and Transfection

The details of plasmids used in this study are presented in **Table** **1**. For the plasmid extraction, the constructed plasmids were induced into 100 µl DH5α‐competent cells by heat shock transformation. Transformed cells were selected on a Luria‐Bertani (LB) ampicillin plate for 12 h at 37 °C. Single colonies were picked and grown in 50 ml fresh LB‐medium supplemented with 100 µg ml^−1^ ampicillin overnight at 37 °C. Plasmid DNA was extracted and purified using TIANprep Mini Plasmid Kit, and confirmed by Sanger sequencing. Finally, plasmid DNA was resuspended in 20 µL nuclease‐free water, and stored at −20 °C. Successful construction of plasmids was determined through fluorescence visualization of transfected cells.

**Table 1:** Detailed information of the key resources, cell lines, recombinant polypeptides and primers in this study.

Reagent or Resource	Source	Identifier
**Chemicals and Materials**		
Poly‐L‐Lysine	Sigma‐Aldrich	Cat# P1274
DnaseI	Sigma‐Aldrich	Cat# DN25
DMEM	Biological Industries	Cat# C11995500BT
OPTI MEM I	Gibco	Cat# 31985070
DMEM/F12	Gibco	Cat# C11330500BT
Glucose‐Free DMEM	Procell	Cat# PM150270
Penicillin/Streptomycin	Biological Industries	Cat# 03‐031‐1B
Trypsin 0.5% EDTA	Biological Industries	Cat# 03‐050‐1A
Dulbecco's phosphate‐buffered saline (DPBS)	Biological Industries	Cat# 02‐023‐1ACS
Fetal Bovine Serum (FBS)	Gibco	Cat# 10099‐141C
Puromycin (for cell sorting)	Yeasen	Cat# 60209ES10
G418	Aladdin	Cat# 108321‐42‐2
Puromycin (for treatment)	MedChemExpress	Cat# HY‐B1743A
Lipoamide	MedChemExpress	Cat# HY‐B1142
Cycloheximide	MedChemExpress	Cat# HY‐12320
Luria‐Bertani	Coolaber	Cat# PM0010
Ampicillin	Sigma‐Aldrich	Cat# A5354
Glutaraldehyde	Servicebio	Cat# G1102
5‐ethyluridine (5EU)	Ribobio	Cat# C00065
Biotin‐azide	Ribobio	Cat# C00101
Azide 488	Beyotime	C0071S‐2
CuSO4	Sigma‐Aldrich	Cat# 451657
Tris(3‐hydroxypropyltriazolylmethyl)amine (THPTA)	Sigma‐Aldrich	Cat# 762342
Aminoguanidine hydrochloride	Sigma‐Aldrich	Cat# 396494
Sodium L‐ascorbate	Sigma‐Aldrich	Cat# 1114
Lithium chloride (LiCl)	Sigma‐Aldrich	Cat# 62476
0.5M EDTA	Invitrogen,	Cat# 15575038
Lithium dodecyl sulfate (LiDS)	Sigma‐Aldrich	Cat# L9781
10% Sodium dodecyl sulfate (SDS)	Invitrogen	Cat# 24730020
DL‐Dithiothreitol (DTT)	Sigma‐Aldrich	Cat# 20‐265
Proteinase K	Invitrogen	Cat# 25530049
EDTA‐free complete protease inhibitor cocktail tablets	Millipore	Cat# 539131
Tris‐HCl PH.7.5	Invitrogen	Cat# 15567027
NaCl	Invitrogen	Cat# AM9760G
MgCl2	Invitrogen	Cat# AM9530G
NP‐40	Thermo Scientific	Cat# 85125
RNase Inhibitor	Thermo Scientific	Cat# EO0382
Triton X‐100	Sangon Biotech	Cat# A600198
Sodium deoxycholate	Sangon Biotech	Cat# A600150
MgOAc	Sigma‐Aldrich	Cat# 63052
KOAc	Sigma‐Aldrich	Cat# 95843
Heparin	Sigma‐Aldrich	Cat# H3149
Urea	Sigma‐Aldrich	Cat# U4883
TRIZOL	Invitrogen	Cat# 10296010CN
Isopropyl‐β‐d‐thiogalactoside (IPTG)	Invitrogen	Cat# AM9464
Tris–HCl pH 8.0	Invitrogen	Cat# 15568025
TEV protease	Invitrogen	Cat# 12575023
GSH	Sangon Biotech	Cat# A600229
Ethanol	Sangon Biotech	Cat# A500737
Linear Acrylamide	Invitrogen	Cat# AM9520
SYBR Green PCR Master Mix	Roche	Cat# 04887352001
Amersham Protran 0.2 µm NC	GE Healthcare Life Science	Cat# 10600001
Tris Buffered Saline buffer (TBS)	Sangon Biotech	Cat# A510025
Tween 20	Sangon Biotech	Cat# A600560
4% Paraformaldehyde	Beyotime	Cat# P0099
Bovine serum albumin (BSA)	Sangon Biotech	Cat# A600903
Saline sodium citrate (SSC)	Thermo Scientific	Cat# 28348
Formamide	Sangon Biotech	Cat# A600211‐0500
Dextran sulfate	Sigma Sigma‐Aldrich	Cat# S4030
tRNA	Roche	Cat# R1753
40% 37.5:1 Arc‐Bis	Sigma‐Aldrich	Cat# 01709
Glycerin	Sigma‐Aldrich	Cat# 1295607
RNase R	Lucigen	Cat #: RNR07250
Ammonium persulfate substitute	Beyotime	Cat# ST005
Tetramethylethylenediamine (TEMED)	Beyotime	Cat# ST728
TBE buffer	Beyotime	Cat# ST723
Western Blot Cell Lysis Buffer	Beyotime	Cat# P0013
GST‐Sepharose columns	GE Healthcare	Cat# GE17‐5130‐01
Protein A/G Magnetic beads	Thermo Scientific	Cat# 88803
RNase I	Thermo Fisher,	Cat# EN0601
T4 RNA Ligase	Thermo Fisher,	Cat# EL0021
HiScript® III Reverse Transcriptase	Vazyme	Cat# R302
VAHTS DNA Clean Beads	Vazyme	Cat# N411
Dynabeads™ MyOne™ Streptavidin T1	Thermo Scientific	Cat# 65602
Centrifugal Filter Unit	Millipore	Cat# UFC901096
Confocal Dishes	Cellvis	Cat# D35‐20‐1.5P
**Critical Commercial Assays**		
TIANprep Mini Plasmid Kit	TIANGEN BIOTECH	Cat# DP103
Lipofectamine 3000 Transfection Reagent	Thermo Scientific	Cat# L3000015
BCA Protein Assay Kit	Thermo Scientific	Cat# 23225
PrimeScript™ RT Reagent Kit	TaKaRa	Cat# RR047A
SDS‐PAGE gels Kit	Beyotime	Cat# P0012A
ExoQuick‐TC Precipitation Solution	System Biosciences (SBI)	Cat# EXOTC10A‐1
PKH26 Red Fluorescent Cell Linker Kit	Sigma‐Aldrich	Cat# PKH26PCL
RNA‐Binding Protein Immunoprecipitation Kit	Sigma‐Aldrich	Cat# 17‐704
Qubit 1X dsDNA HS	Thermo Scientific	Cat #: Q33230
Epi^TM^ mini longRNA‐seq kit.	Epibiotek	Cat# E1802
**Cell line, Vector and Recombinant Protein**		
E. coli DH5α	Sangon Biotech	Cat# B528413
E. coli BL21(DE3)	Sangon Biotech	Cat# B528419
Sh‐SY5Y Cells	ATCC	Cat# CRL‐2266
FUS/TLS Double Nickase Plasmid (h)	Santa Cruz	Cat# sc‐400612‐NIC
pCT‐CD63‐GFP	System Biosciences (SBI)	Cat# CYTO120‐PA‐1
pLV[Exp]‐mCherry/Neo‐EF1A>FLAG/FUS/TLS (Full length FUS)	VectorBuilder	Designed in this study
pLV[Exp]‐mCherry/Neo‐EF1A>{FLAG/FUS/TLS (Del32aa)} (Mutation FUS)	VectorBuilder	Designed in this study
pLV[Exp]‐EF1A>FUS/TLS:mCherry‐CMV>Neo	VectorBuilder	Designed in this study
FUS recombinant polypeptide 1 (1‐526 aa)	MerryBio	Designed in this study
FUS recombinant polypeptide 2 (50‐400 aa)	Proteintech	Cat# Ag2150
FUS recombinant polypeptide 3 (1‐380 aa)	MerryBio	Designed in this study
FUS recombinant polypeptide 4 (280‐375 aa)	MerryBio	Designed in this study
FUS recombinant polypeptide 5 (380‐526 aa)	MerryBio	Designed in this study
**Instruments and Software**		
NanoDrop 2000	Thermo Scientific	2000
Real‐Time PCR Detection System	Bio‐Rad	CFX96 Touch
Laser image Scanner	Typhoon	FLA 9500
Confocal Laser Scanning Microscopy	Zeiss	LSM888
Gel Imaging System	Tanon	2500
Transmission Electron Microscopy	JEOL	JEM‐1400Flash
Nanosight NS300	Malvern	NS300
Hypoxia Chamber Incubator	HUAYI NINGCHUANG	Smartor 118
CFX Manager Software	Bio‐Rad	Version 3.1
TapeStation 2200	Agilent	2200
Qubit 2.0	Life Technologies	2.0
HiSeq 2500	Illumina	2500
Image‐J Software	National Institutes of Health	N/A
GraphPad Prism Software	GraphPad	version 6
Zen Blue Edition software	Zeiss	Version 2.3
DigitalMicrograph Software	Gatan	Version 3.9
PyMOL Software	DeLano Scientific LLC	N/A
SPSS software	IBM	Version 27.0
Fastp software	N/A	N/A
Hisat2 software	N/A	Version 2.1.0
**Primer and Probe Sequences**		
CircOGDH (hsa_circ_0003340)‐ Forward	AACGGATTTGGTCGTATTGGG	
CircOGDH (hsa_circ_0003340)‐ Reverse	TGAGTCTCCAATGGCAACCC	
CircMap2 (hsa_circ_0057995)‐ Forward	GACAGAGAAACAGCAGAAAG	
CircMap2 (hsa_circ_0057995)‐ Reverse	GCCTTGTTATTTCTCCTGCA	
18s‐ Forward	ACACGGACAGGATTGACAGA	
18s‐ Reverse	GGACATCTAAGGGCATCACA	
GGUG Oligo	UUGUAUUUUGAGCUAGUUUGGUGAC‐Cy3	
CircOGDH Probe Mix	Designed by RiboBio, Cat# lnc1031978	
CircMap2 Probe Mix	Designed by RiboBio, Cat# lnc1031885	

For the plasmid transfection, SH‐SY5Y cells were seeded in 6‐well plates and allowed to reach 70% confluence prior to transfection. Plasmids were transfected with Lipofectamine 3000 according to manufacturer's Transfection Protocol. Briefly, 5 µg of plasmid was diluted with 125 µL of Opti‐MEM supplemented with 10 µL of P3000 reagent. At the same time, 3.75 µL of lipo3000 was diluted with 125 µL of Opti‐MEM. The diluted plasmid was added to the diluted lipo3000 and incubated for 15 min at room temperature. The DNA‐lipid complexes were then added to cells, which were incubated at 37 °C for 72 h before analysis.

### Establishment the Stable Cells Lines

The details of mCherry‐FUS and EGFP‐CD63 lentivirus and FUS‐KO Double Nickase Plasmid (h) used in this study are presented in Table 2. When SH‐SY5Y cells were grown to 60%−70% confluence, the mCherry‐FUS and EGFP‐CD63 lentivirus were added into medium for 3 days culture, and then cells were selected with 600 µg ml^−1^ G418 and 2.5 µg ml^−1^ Puromycin for 7 days to generate the double labeled stable cells lines. Similar, the FUS/TLS double nickase plasmid was transfected into SH‐SY5Y cells, selected by 2.5 µg ml^−1^ Puromycin to generate the FUS knockout cell line. The successful construction of mCherry‐FUS/EGFP‐CD63 stable cell line and FUS‐knockout stable cell line were sorted on a BD FACSAria III flow cytometer before expending and confirmed by western blotting detection.

### Hypoxia Cell Model and Drugs Treatment

The hypoxia cell model in this study was established by oxygen–glucose deprivation (OGD) manner.^[^ ^11^ ^]^ The SH‐SY5Y cells seeded into 10 cm dishes and grown to 80% confluence. Washing the cells with PBS and adding the glucose‐free deoxygenated DMEM into the plate. Then, cells were culture in the hypoxia chamber incubator with 95% N_2_ and 5% CO_2_ for 3 h as the OGD modeling. After that, replacing culture medium with DMEM and incubating the cells in the normal chamber with 95% air, 5% CO_2_ as reperfusion. Control groups were incubated with DMEM under normal condition for the same time. For the drugs treatment groups, the compounds were diluted to appropriate concentrations with the modeling and normal medium and added into plate at the beginning of OGD.

### sEVs Isolation and Characterization

SH‐SY5Y cells were cultured in 10 cm culture dishes and used for sEV production when cell density reached 80%−90%. 80 ml of cell supernatant from 8 culture dishes was collected for each group. For the control group sEV production, cells were washed twice with PBS and further cultured in DMEM without FBS for 24 h. For hypoxia and reperfusion sEV production, the cells were washed twice with PBS and subjected to 3 h of OGD; subsequently, the culture medium was replaced with DMEM without FBS for continued culture as reperfusion, and sEVs were collected at the designated time‐points. Hypoxia (H3h/R0h) sEVs were extracted from the hypoxic medium without reperfusion. The culture medium of each group was collected and centrifuged at 300 g for 10 min to remove cells, at 2000 g for 20 min to remove cell debris and apoptotic bodies. Next, the supernatant would be passed through a 0.22‐micron filter and further concentrated to 1 ml using centrifugal filter tubes with centrifugation speed of 3220 g for 15 min. The concentrated liquid was incubated with ExoQuick‐TC Precipitation Solution overnight according to the instructions, and the sEV pellet were obtained the next day by centrifugation at 1500 g twice. The pellet was re‐suspended in PBS and used for the following analysis.

For the sEVs characterization, 10 µl fresh sEVs were dripped on a carbon‐coated copper grid and allowed for 10 min to dry, and then fixed with 2.5% glutaraldehyde for 5 min. Next, the fixed grid was washed three times by PBS. Finally, sEV micrographs were captured through Transmission Electron Microscopy (TEM) with voltage setting at 120 Kv. Nanoparticle Tracking Analysis (NTA) was used to measure sEV concentration and particle size distribution by Nanosight NS300. After rinsing the sample chamber with PBS, 500 µl of sEV suspension diluted in PBS was added to the sample chamber for analysis. Western blotting was used to detect sEV classical markers, such as CD63 and calnexin was used as a negative control.

### sEV Labeling and Uptake Experiment

To visualize sEV's internalization and distribution in recipient cells, sEVs were labeled with PKH67/PKH26 fluorescent dye using a PKH67/PKH26 fluorescent cell linker kit. According to the instruction manual, 20 µl sEV suspension was diluted with 500 µl Diluent C, and then the mixture of 500 µl Diluent C and 2 µl dye was added into sEV solution to incubate for 5 min. Subsequently, 10 ml DMEM complete medium without sEVs (DMEM+ 10% sEVs‐free FBS) was added to the reaction system to remove excess dye. Thereafter, the sEVs were retrieved by means of centrifugal filter tubes, and subjected to two rounds of washing with 10 ml of DMEM complete medium without sEVs and two further rounds of washing with PBS.

To observe the uptake of different sEVs, 20 µl of sEVs was used harvested from 4 10 cm‐dishes cells in each group for sEV labeling. After the OGD treatment of cells, the culture medium was changed to normal medium, and equal amounts of sEVs were added simultaneously. After co‐culture for corresponding periods, cells were fixed with 4% PFA and performed the subsequent fluorescence staining steps. The images were captured by the confocal laser scanning microscopy (CLSM) using a Zeiss LSM888. The fluorescence quantification was performed using Zen v2.3 software.

### Nuclear and Cytoplasmic Separation

SH‐SY5Y cells were seeded into 10 cm dishes and grown to 80% confluency. Cell culture media was replaced by the fresh glucose‐free DMEM without fetal bovine serum. Following stress with 3 h hypoxia, the media was aspirated and cells were washed with PBS for two times. Cells were harvested by scrapers and lysed in 0.1 mL lysis buffer 1 (50 mm Tris‐HCl pH 7.4, 20 mm NaCl, 1 mm MgCl_2_, 0.25 mm DTT, 20 U mL^−1^, 1% Cocktail protease inhibitor) on ice for 10 min. After 20 s vertexing, added equal volume of lysis buffer 2 (50 mm Tris‐HCl pH 7.4, 0.1% NP‐40, 0.25 mm DTT, 20 U mL^−1^ RNase Inhibitor, 1% Cocktail protease inhibitor) on ice for another 10 min. Then, vertexing the lysate for 20 s and spun at 3500 g for 15 min, supernatant (cytoplasm) and precipitate (nuclear) were harvested, respectively. The cytoplasm sample was obtained from the supernatant after spinning at 12 000 rpm for 10 min. The precipitate was resuspended in 0.5 mL lysis buffer 3 (50 mm Tris‐HCl pH 7.4, 150 mm NaCl, 1 mm MgCl2, 0.25 mm DTT, 0.5% NP‐40, 20 U mL^−1^ RNase Inhibitor, 1% Cocktail protease inhibitor) and spun at 3500 g for 15 min. Repeat this step for three times to obtain nuclear precipitation. The pellet was further lysed by the 0.1 mL RIPA lysis buffer (50 mm Tris‐HCl pH 7.4, 150 mm NaCl. 1% Triton X‐100, 1% sodium deoxycholate, 0.1% SDS, 0.25 mm DTT, 0.5% NP‐40, 20 U mL^−1^ RNase Inhibitor, 1% Cocktail protease inhibitor) on ice for 10 min. The nuclear sample was finally harvested from the pellet lysate after 12 000 rpm for 10 min. The cytoplasm and nuclear samples mixed with the 5 x SDS PAGE loading buffer and boiled at 100°C for western blot analysis. Adding 1 mL Trizol lysis buffer into those for RNA extraction and detection.

### Purification of Stress Granule Cores

The isolation of SGs cores was adapted from two papers.^[^ ^14^, ^58^ ^]^ SH‐SY5Y cells were grown to 80% confluency. After stressing under hypoxic conditions with OGD model, cells were washed with PBS for two times and harvested by scraper. The cell pellet was lysed in 1 mL SGs lysis buffer (50 mm Tris‐HCl pH 7.4, 2 mm MgOAc, 100 mm KOAc, 50 µg mL^−1^ heparin, 0.5 mm DTT, 0.5% NP‐40, 10 U mL^−1^ RNase Inhibitor, 1% Cocktail protease inhibitor) for 15 min on ice, and spun at 4 °C for 5 min at 1000 g. Protein concentration was quantified using BCA Protein Assay Kit according to manufacturer's instructions. Five hundred micrograms of total protein was bound to 20 µl no‐coating Protein A/G Dynabeads beads and nutated at 4 °C for 30 min. After discarding the beads, 2.5 µg anti‐G3PB1 antibody and 20 µl Protein A/G Dynabeads beads were added to the supernatant and nutated at 4 °C for 12 h. Dynabead were then washed in 1 mL wash buffer 1 (20 mm Tris‐HCl pH 8.0, 200 mm NaCl) for 3 × 2 min, 1 mL wash buffer 2 (20 mm Tris‐HCl PH 8.0, 500 mm NaCl) for 1 × 2 min, 1 mL wash buffer 3 (50 mm Tris‐HCl PH 8.0, 2 mm MgOAc, 100 mm KOAc, 50 µg mL^−1^ heparin, 2 m Urea) for 1 × 2 min. Following the final wash, Dynabeads were then resuspended in 2X SDS PAGE loading buffer and boiled at 100 °C for 10 min for western blot detection, and mixed with 1 mL TRIZOL reagent into those for RNA extraction and detection.

### Purification of Recombinant Protein

Protein expression and purification were synthesized by MerryBio Co., Ltd and Proteintech Co., Ltd (Table 2), and carried out following the standard protocol. Briefly, the FUS peptides plasmids were fused to pGEX‐4T‐1 and expressed in bacteria BL21 (DE3). After induction with 0.2 mm isopropyl‐β‐d‐thiogalactoside (IPTG), cultivation was continued at 15 °C overnight. Cells were harvested by centrifugation and washed three times with cold PBS. The cells were lysed with lysis buffer (50 mm Tris–HCl pH 8.0, 100 mm NaCl, 1 mm DTT, 1% Cocktail protease inhibitor) containing 1 mg ml^−1^ lysozyme by sonication on ice. GST‐Sepharose columns (1 mL) pre‐equilibrated with 20 mL TEV protease cleavage buffer (10 mm Tris‐HCl pH 8.0, 150 mm NaCl, 0.1% NP‐40, 1 mm DTT) was added to the supernatant and rotated at 4 °C for 2 h. The beads were then washed three times with TEV protease cleavage buffer and the recombinant protein was eluted from the resin by incubation overnight at 4 °C with 10 µg mL^−1^ TEV protease. This was done to cleave the desired protein from the GST tag, which was still bound to the GST‐Sepharose resin after the overnight cleavage reaction. The target proteins were then eluted with 2–5 mL of elution buffer (50 mm Tris‐HCl, pH 8.0, 40 mm reduced glutathione) with rotation for 5 min at room temperature. Finally, fractions from the flow‐through, washes and elutes were analyzed by SDS‐PAGE.

### Western Blot Analysis

Cells were homogenized by western cell lysis buffer supplemented with 1% Cocktail protease inhibitor. After quantification by BCA assay kit, equal amounts of protein were loaded onto SDS‐PAGE gels. Cell lysate was fractionated on 8%, 10%, or 12% SDS‐PAGE according to their molecular weight, and subsequently transferred to 0.22 µm NC membrane. The membranes were blocked with 5% milk, washed with TBST (TBS supplemented with 0.1% Tween 20), and incubated with primary antibody in sequence. After incubation with primary antibody at 4°C overnight (**Table** **2**), the membrane was washed with TBST and incubated with HRP‐conjugated secondary antibody at room temperature for 1 h. Finally, the protein bands were visualized using a Tanon 2500 gel imaging system and analyzed by Image‐J Software. All target band values were normalized to β‐actin, and the fold changes were calculated as the relative quantity compared to the control.

**Table 2:** Detailed information of the primary antibodies in this study.

Antibody name	SOURCE	IDENTIFIER	RRID	Application
FUS/TLS	Abcam	Cat# ab124923	AB_10972861	IF: 1:200
	Santa Cruz	Cat# sc‐47711	AB_2105208	WB: 1:200
G3BP	Abcam	Cat# ab181150	AB_2847886	WB: 1:1000 IF: 1:200
	Abcam	Cat# ab56574	AB_941699	IF: 1:200
CD63	Abcam	Cat# ab217345	AB_2754982	IF: 1:200
				WB: 1:1000
Calnexin	Cell Signaling Technology	Cat# 2679	AB_2228381	WB: 1:1000
GAPDH	Santa Cruz	Cat# sc‐47724	AB_627678	WB: 1:200
Histone H3	Abcam	Cat# ab1791	AB_302613	WB: 1:1000
Flag	Proteintech	Cat# CL647‐66008	AB_2920272	WB: 1:1000
Peroxidase AffiniPure Goat Anti‐Mouse IgG(H+L)	Yeasen	Cat# 33201ES60	AB_10015289	WB: 1:5000
Peroxidase AffiniPure Goat Anti‐Rabbit lgG(H+L)	Yeasen	Cat# 33101ES60	AB_2922405	WB: 1:5000
Goat Anti‐Mouse IgG H&L (Alexa Fluor® 405)	Abcam	ab175660	AB_2885184	IF: 1:200
Goat Anti‐Rabbit IgG H&L (Alexa Fluor® 594)	Abcam	ab150080	AB_2650602	IF: 1:200
Donkey Anti‐Rabbit IgG H&L (Alexa Fluor® 488)	Abcam	ab150073	AB_2636877	IF: 1:200

### Immunofluorescence and Living Cells Images

Cells were immobilized using a 4% paraformaldehyde for 15 min. Following this, the cells were washed with PBS and permeabilized with 0.5% Triton X‐100 for 15 min. Subsequently, the cells were washed with PBS and subjected to a blocking step using a 5% bovine serum albumin (BSA) solution for 1 h at room temperature. The cells were then incubated overnight at a temperature of 4° C with a primary antibody (Table 3). On the subsequent day, the cells were washed with PBS and incubated with the secondary antibodies for 1 h. The images were acquired using the confocal laser microscopy Zeiss LSM888. The fluorescence quantification was performed using Zen v2.3 software.

The mCherry‐FUS/EGFP‐CD63 double labeled cells were seeded in 3.5 cm confocal dishes and grown to 80% confluency. Then, the culture dish was placed in a live cell workstation under hypoxic condition with 95% N_2_ and 5% CO_2_ for 3 h dynamic observation by using the confocal laser microscopy Zeiss LSM888. The images were taken every 20 min during the periods.

### Fluorescence In Situ Hybridization (FISH)

The Cy3 labeled circRNA probe mixes were set complementary to the junction reads of target CirRNA, and purchased from RiboBio Co., Ltd. The FUS‐specific GGUG probe was cited from previous study^[^ ^59^ ^]^ (Table 2). SH‐SY5Y cells were seeded on a 35 µm glass bottom dish and grown to 80% confluency. Cells were treated as indicated, followed by a quick wash with PBS for two times and fixed by 4% paraformaldehyde for 10 min at room temperature. After that, cells were permeabilized by 70% ethanol for 30 min and 0.3% Triton X‐100 in PBS for 5 min at 4 °C. Subsequently, the cells were washed with 2x saline sodium citrate (SSC) for three sets of 5 min. The dish was then incubated with pre‐hybridization solution (consisting of 40% formamide, 10% dextran sulfate, 0.25 µg mL^−1^ tRNA, 10 U mL^−1^ RNase Inhibitor in 2x SSC) at 37 °C in a humidified chamber for 1 h. Next, the cells were incubated with hybridization solution (containing 40% formamide, 10% dextran sulfate, 0.25 µg mL^−1^ tRNA, 1 µm FISH probes, 10 U mL^−1^ RNase Inhibitor in 2x SSC) for 12 h at 37 °C without washing. Following the hybridization step, the cells were washed with wash buffer 1 (consisting of 40% formamide, 0.3% Tween‐20 in 2x SSC) for 5 min at 37 °C, wash buffer 2 (4x SSC) for 5 min at 37 °C, and wash buffer 3 (2x SSC) for 5 min at 37 °C. Protein staining was performed according to the “immunofluorescence” section, BSA blocking, mounting, and the samples were imaged using a confocal microscope.

### RNA Immunoprecipitation (RIP)

The RIP assay was performed using the RNA‐Binding Protein Immunoprecipitation Kit and followed by the previously studies.^[^ ^60^, ^61^ ^]^ Briefly, SH‐SY5Y cells were lysed using rip lysate on ice for 30 min. Protein A/G magnetic beads were incubated with antibodies specific to FUS (Table 3) for 1 h at 4 °C, forming a magnetic bead‐antibody complex. The complex was added to the cell suspension and incubated overnight at 4 °C with gentle rotation. The RNA molecules bound to the RNA‐binding protein (RBP) were immobilized using a magnet, while the unbound materials were removed by washing with a washing buffer. The RNAs were then purified using the proteinase K buffer, followed by quantitative analysis using RT‐qPCR.

### RNA Extraction, RNase R Treatment, and RT‐qPCR Detection

Total RNA from cells and sEVs samples was extracted using the TRIZOL reagent, following the manufacturer's instructions. Briefly, cells and sEVs were washed with PBS and then lysed in 1 mL of TRIZOL reagent. Phase separation was achieved by adding chloroform, and the organic phase supernatant containing the RNA was collected after centrifugation at 12,000 rpm for 10 min. Isopropanol was used to precipitate the RNA at 4 °C, and the RNA pellet was washed with 75% ethanol. Linear acrylamide in isopropanol was added to aid in RNA precipitation. Finally, the RNA was resuspended in RNase‐free water, and the concentration was determined using a NanoDrop spectrophotometer.

For circRNA enrichment, after the total RNA extraction, the linear RNA was removed by incubating the sample for 15 min at 37 °C with 3 U µg^−1^ of RNase R, which was an enzyme that specifically degrades linear RNA but not circRNA. This step was performed to enrich the circRNAs. Subsequently, the concentration was quantified using the Qubit 1X dsDNA HS assay.

For reverse transcription, cDNAs were synthesized using the PrimeScript RT Reagent Kit with gDNA Eraser, following the manufacturer's instructions. Five hundred nanograms of RNA of each sample was used for the reverse transcription. SYBR Green PCR Master Mix was used for the RT‐qPCR detection. The total mixture was set up in 20 µL consisting of 10 µL master mix, 1 µL 10 nm forward primers, 1 µL 10 nm reverse primers, 6 µL H_2_O, and 2 µL cDNA mixes (1:3 dilution). The reaction ran on the CFX96 Touch Real‐Time PCR Detection System by the cycling conditions: 95 °C for 10 min for polymerase activation, followed by 40 cycles of 95 °C for 10 s denaturation and 60 °C for 20 s annealing and extension. The relative expression of each mRNA was analyzed by CFX Manager Software using the comparative Ct (2^ΔΔ^Ct) method, and normalized relatively to β‐actin or 18s expression. The primer sequences used in this study are presented in Table 2.

### 5EU‐Click Chemistry and Nascent RNA Extraction (for RICK‐seq)

The 5EU‐Click Chemistry for newly nonspecific RNAs labeling and nascent circRNAs sequencing (RICK‐seq) method were carried out as described previously.^[^ ^22^ ^]^ In brief, primary neurons or SH‐SY5Y cells were seeded into 10 cm dishes and treated with 0.25 mm EU in the culture medium for 16 h before following operations.

For nonspecific RNA labeling, after modeling under stress conditions, the cells were fixed with 4% paraformaldehyde for 15 min and permeabilized with 0.5% Triton X‐100 for another 15 min at room temperature. Then, the fluorescein‐labeled azide was linked to the 5EU‐labeled intracellular RNAs by incubating with the click reaction solution containing 0.25 mm biotin‐azide, 0.3 mm CuSO4, 0.6 mm THPTA, 0.1 mm aminoguanidine, and 5 mm sodium L‐ascorbate for 5 min at room temperature. The reaction was stopped by washing the cells with 2 mm EDTA for three sets of 3 min. Protein staining was performed following the immunofluorescence steps, from BSA blocking to mounting, and the samples were imaged using a confocal microscope.

For the nascent RNA extraction (for RICK‐seq), cells were fixed with 90% ethanol on ice for 30 min after modeling. The cells were then permeabilized with 0.5% Triton X‐100 for 15 min on ice, and the biotin labeled azide was linked to the 5EU‐labeled RNAs by incubating with the click reaction solution containing 0.25 mm biotin‐azide, 0.3 mm CuSO4, 0.6 mm THPTA, 0.1 mm aminoguanidine, and 5 mm sodium L‐ascorbate for 5 min at room temperature. Next, the cells were washed with 2 mm EDTA to stop the reaction, and the cell lysate was harvested using the lysis buffer (20 mm Tris‐HCl, pH 7.5, 500 mm LiCl, 1 mm EDTA pH 8.0, 0.5% lithium‐dodecylsulfate, and 5 mm DTT, 10 U ml^−1^ RNase inhibitor). The cell lysate was mixed with streptavidin‐conjugated magnetic beads to isolate the 5EU‐labeled RNAs. Finally, library preparation and sequencing samples were obtained following the proteinase K buffer incubation and RNA extraction steps.

### High‐Throughput RNA Sequencing and Bioinformatics Analysis

The RNA library preparation and high‐throughput RNA sequencing were performed by Epibiotek Co., Ltd following the standard protocol. The RNAs were fragmented to ≈200 nucleotides and subjected to first‐strand and second‐strand synthesis, adaptor ligation, and low‐cycle amplification using the Epi mini longRNA‐seq kit. The purified library products were evaluated using the Agilent 2200 TapeStation and Qubit 2.0, and then diluted to 10 pM for cluster generation followed by high‐throughput sequencing on the HiSeq 2500 platform. The raw read data from each sample were processed using the fastp software. Raw data mapping was performed using Hisat2 software, and HTseq was used to calculate the number of reads mapped to the genome. The expression data were standardized using FPKM.

The unique and common parts between two subsets of circRNA (Log2FC>2) were analyzed and visualized using the “ggplot2” and “VennDiagram” packages. Volcano Plots were generated using the “ggplot2” package based on the differential expression analysis, with the color determined by the filtering criteria. GO enrichment analysis and visualization were conducted using the “clusterProfiler” and “ggplot2” packages.

### Enhanced Cross‐Linking and Immunoprecipitation Sequencing (eCLIP‐seq) and Motif Analysis

Cells were cross‐linked in UV cross‐linking (254 nm, 400 mJ cm^−2^) to fix RNA binding protein (RBP)–RNA. The cell pellet was washed twice with 1×PBS and then lysed in iCLIP buffer (25 mm Tris pH 7.4, 150 mm KCl, 5 mm EDTA, 0.2% CA‐630, 0.05% SDS) at 4°C. The cell lysate containing RNA binding protein (RBP)–RNA complexes was sheared by ultrasonication. Limited digestion was performed with RNase I. Then centrifuged to obtain the supernatant, one‐tenth of which was aliquoted as input. RBP‐RNA complexes were immunoprecipitated with FUS antibodies (10 µg) with Dynabeads Protein A, and strictly washed. Cut off the membrane of the target protein by running the SDS‐PAGE gel and transferring the membrane, and digested with proteinase K to isolate RNA. A second DNA adapter, incorporating a 10‐random base sequence at the 5′ end, was ligated to the cDNA fragment. Cleanup and PCR amplification ensued before size selection. The libraries were purified by purification beads and analyzed on Agilent 2100 Bioanalyzer for measuring fragment size distribution (≈150 bp). The qualified libraries were sequenced on Illumina Nova Seq 6000.

For paired‐end data, scripts were run twice, with Fastq files subjected to quality control. Adapters were removed by Cutadapt, and reads were mapped to the human reference genome (hg38) via STAR. Fractionated data underwent series conversion with Bowtie‐align‐s. Alignment SAM files were sorted using Samtools, and a custom script facilitated PCR duplicate removal. Merging of technical replicates preceded downstream analysis. Peak‐calling was executed using MACS2, and peak annotation and motif analysis were performed using Homer2. IDR output was utilized for QC checks. Differential expression analysis utilized DEseq2, retaining data with |log2Foldchange| > 1 and p‐value < 0.05 for subsequent analysis. Input samples aided in background noise removal during peak calls. The eCLIP‐sequencing and analysis service were provided by DIATRE Biotechnology(China).

### Electrophoretic Mobility Shift Assay (EMSA)

The oligonucleotide probes used were identical to those in the “FISH” section. A mixture of 100 nm RNA probes and purified protein peptides or cell cytoplasmic lysates in 1 mL binding buffer containing 10 mm Tris‐HCl pH 8.0, 10 mm NaCl pH 8.0, 25 mm KCl, 1 mm MgCl2, 1 mm DTT, and 1 mg mL^−1^ tRNA was incubated at room temperature for 30 min. The samples were then resolved through 4% native acrylamide gels (0.9 mL 40% 37.5:1 Arc‐Bis, 0.5 mL 50% glycerin, 0.2 mL 10% ammonium peroxodisulphate, 0.01 mL tetramethylethylenediamine in 10 mL 1xTBE buffer) under an electric field of 15 V cm^−1^ for 40 min. The gels were subsequently visualized with a Typhoon FLA 9500 laser scanner.

### Molecular Modeling

The molecular structure of FUS was obtained from the AlphaFold Protein Structure Database (Model ID: AF‐P35637‐F1).^[^ ^62^, ^63^ ^]^ After removing the nucleic acid molecules, the Zf_RanBP domain structure was generated from a crystal structure (PDBID: 6G99). The circRNAs predictions were obtained by sequentially constructing secondary and tertiary structures based on their own sequences (the details sequences of those circRNAs were described in Table 3). The secondary structures of RNA were analyzed using RNAfold (http://rna.tbi.univie.ac.at/cgi‐bin/RNAWebSuite/RNAfold.cgi) with the “minimum free energy (MFE) and partition function” selection in the folding algorithm and the “assume RNA molecule to be circular” option in the advanced parameters. The outcomes were further input into 3dRNA/DNA (http://biophy.hust.edu.cn/new/3dRNA)^[^ ^64^ ^]^ with the “circular RNA” option and “Default” procedure. The prediction of FUS‐circRNAs complex structures was performed by HADDOCK (https://wenmr.science.uu.nl/haddock2.4),^[^ ^65^ ^]^ and the Zf_RanBP‐circRNAs complex structures were analyzed by Z‐DOCK (http://zdock.umassmed.edu).^[^ ^66^ ^]^ The RNA‐proteins docking analysis was performed using the default settings without blocking any residues. Visualization of complexes and local high‐resolution images were performed using Biorender and PyMOL.

**Table 3:** Sequence information of four candidate CircRNAs in this study.

CircRNA Name	Sequence
CircOGDH hsa_circ_0003340	TCATGGGACATTTTTTTTCGCAACACGAATGCCGGAGCCCCACCGGGCACTGCCTACCAGAGTCCCCTTCCCCTGAGCCGAGGCTCCCTGGCTGCTGTGGCCCATGCACAGTCCCTGGTAGAAGCACAGCCCAACGTGGACAAGCTCGTGGAGGACCACCTGGCAGTGCAGTCGCTCATCAGGGCATATCAGATACGAGGGCACCATGTAGCACAGCTGGACCCCCTGGGGATTTTGGATGCTGATCTGGACTCCTCCGTGCCCGCTGACATTATCTCATCCACAGACAAACTTG
CircOGDH mmu_circ_0000231	TCATGGGACATTTTTTTCCGAAACACCAATGCTGGAGCCCCACCGGGCACTGCCTACCAGAGCCCCCTTTCCCTGAGTCGAAGCTCCCTGGCTACCATGGCCCATGCACAGTCCCTGGTGGAAGCACAACCTAACGTCGACAAACTCGTGGAGGACCACTTGGCGGTGCAGTCTCTCATCAGGGCATATCAGATACGAGGGCACCATGTAGCACAGCTGGACCCCCTGGGGATTTTGGATGCTGATCTGGACTCCTCCGTGCCCGCTGACATTATCTCATCCACAGACAAACTTG
CircMAP2 hsa_circ_0057995	ATTCTTCAGCTTGTCTCTAACCGAGGAAGCATTGATTGGGAGCTACTCATTCAGAAAATTAAAAGAAAGAAGCCAGAAAATATTATCAACCCTTTGAGAACACGACACAACGAACTTTATATTTTACCACTTCCTTGAATAGTTGCAGGAGAAATAACAAGGCATTGAAGAATGGCAGATGAACGGAAAGATGAAGCAAAGGCACCTCACTGGACCTCAGCACCGCTAACAGAGGCATCT GCACACTCACATCCACCTGAGATTAAGGATCAAGGCGGAGCAGGGGAAGGACTTGTCCGAAGCGCCAATGGATTCCCATACAGGGAGGATGAAGAGGGTGCCTTTGGAGAGCATGGGTCACAGGGCACCTATTCAAATACCAAAGAGAATGGGATCAACGGAGAGCTGACCTCAGCTGACAGAGAAACAGCAGAGGAGGTGTCTGCAAGGATAGTTCAAGTAGTCACTGCTGAGGCTGTAGCAGTCCTGAAAGGTGAACAAGAGAAAGAAGCTCAACATAAAGACCAGACTGCAGCTCTGCCTTTAG
CircMAP2 mmu_circ_0008896	AAAGAAGCCAGAACATACCACCAGCCGTTTGAGAATACAACATAGCAAACTTCACTACTTTACAACTTCCTTGAATTGGCGACAGTACAGAGATCTGAAAGATGGCCGACGAGCGGAAAGATGAAGGAAAGGCACCACACTGGACATCAGCCTCACTCACAGAGGCAGCTGCACACCCTCACTCTCCAGAGATGAAGGACCAGGGTGGGGCAGGGGAAGGGCTGAGCCGCAACGCCAATGGATTTCCATACAGAGAGGAGGAGGAAGGCGCCTTTGGGGAGCACAGGTCACAGGGCACCTATTCAGATACCAAAGAGAACGGGATCAACGGAGAGCTGACCTCAGCTGACAGAGAAACAGCAG
CircRims2 hsa_circ_0135404	AAAGCTGCACCAACAATTTGAAATGTATAAGGAGCAAGTCAAGAAGATGGGAGAGGAATCGCAGCAGCAGCAAGAGCAGAAGGGTGATGCCCCGACCTGTGGCATCTGCCACAAGACAAAATTTGCAGATGGATGCGGCCATAATTGTTCCTATTGCCAAACCAAGTTCTGTGCTCGATGTGGAGGTCGAGTGTCTTTACGCTCAAACAAG
CircRims2 mmu_circ_0005631	AAAGCTGCACCAACAATTTGAAATGTATAAGGAGCAAGTCAAGAAGATGGGAGAGGAATCGCAGCAGCAGCAAGAGCAGAAGGGTGATGCCCCGACCTGTGGCATCTGCCACAAGACAAAATTTGCAGATGGATGCGGCCATAATTGTTCCTATTGCCAAACCAAGTTCTGTGCTCGATGTGGAGGTCGAGTGTCTTTACGCTCAAACAAG
Circ Slc39a7 (Mouse, no match)	GCACTGGGGGCCACAGTGCTGATCTCCGCAGCTCCGTTCTTCGTGCTGTTCCTCATCCCAGTAGAATCTAACTCTCCCAGGCACCGCTCTCTGCTCCAGATCCTGCTCAGTTTTGCTTCCGGGGGGCTCCTGGGTGATGCGTTCCTCCACCTCATCCCGCATGCCTTGG
Circ Slc39a7 (Human, no match)	N/A
CircTlk1 hsa_circ_0057037	GTGCAATGGATGAGCTTCATAGTCTGGATCCAAGAAGGCAAGAGTTATTGGAAGCTAGATTTACTGGAGTTGCAAGTGGGAGCACTGGAAGTACGGGCAGTTGCAGTGTTGGAGCTAAA
CircTlk1 mmu_circ_0010155	GTGCAATGGATGAGCTTCACAGTCTGGACCCAAGAAGGCAAGAATTATTGGAAGCAAGATTTACTGGAGTTGCTACTGGGAGTACTGGGAGTACTGGGAGTTGCAGTGTTGGAGCAAAA

### Statistics Analysis

The intensity of immunofluorescence was measured using Plot profile tool of ImageJ software; Each band signal of target proteins was initially measured using ImageJ software, followed for normalization of internal reference proteins (GAPDH), which data from each group were further normalized against the mean values of the control group for the final statistical analysis; The comparative CT method referred to as the 2^−ΔΔCT^ method, a widely used method to present relative gene expression was used in this study. Relative gene expression in each group were normalized by internal control 18S and then compared with that in corresponding control group. All statistical analyses were performed using SPSS software (version: 22.0), and data visualization was conducted using GraphPad Prism 8.01 software. Student's two‐sample t‐tests were used to compare values between two groups. For comparisons involving two more groups, One‐way analysis of variance (one‐way ANOVA) with Tukey's or Dunnett's post hoc tests to adjust for multiple comparisons. The data were presented as mean ± standard deviation (SD). p‐value of less than or equal to 0.05 was considered statistically significant. Representative images were chosen without bias and exhibited typical features consistent with the data or overall trends.

## Conflict of Interest

The authors declare no conflict of interest.

## Author Contributions

J.Z., Y.W., X.S., and K.C. contributed equally to this work. A.X., D.L., and H.M. initiated the project and designed the experiments. J.Z. performed most of the experiments and data analysis. Y.W., X.S., K.C., M.K., N.H., Z.T., and M.P. provided valuable assistance with cell culture, sEVs isolation, RNA detection, and RIP procedures. J.Z., Y.W., X.S., and K.C. wrote the manuscript. D.L., H.M., and A.X. supervised the whole study and approved the final version. S.Z., J.Z., W.H., and H.Z. provided additional guidance and suggestions for this project. The manuscript is attributed to contributions from all authors.

## Supporting information

Supporting Information

## Data Availability

The data that support the findings of this study are available from the corresponding author upon reasonable request.
