# Supplementary material for: FUS Selectively Facilitates circRNAs Packing into Small Extracellular Vesicles within Hypoxia Neuron
Source: Adv Sci (Weinh). 2024 Jun 26;12(26):2404822. doi: 10.1002/advs.202404822 (PMC12245079; doi:10.1002/advs.202404822)
Supplement: Supplementary file 1 — Supporting Information [file ADVS-12-2404822-s001.pdf]

ADVANCED SCIENCE
Open Access

Supporting Information

for *Adv. Sci.*, DOI 10.1002/advs.202404822

FUS Selectively Facilitates circRNAs Packing into Small Extracellular Vesicles within Hypoxia Neuron

Jiankun Zang, Yousheng Wu, Xuanlin Su, Kaiwei Cai, Man Ke, Niu He, Huili Zhu, Zefeng Tan, Jielin Zhu, Wensheng He, Min Peng, Shiqing Zhang, Hongcheng Mai, Anding Xu* and Dan Lu**

Figure S1

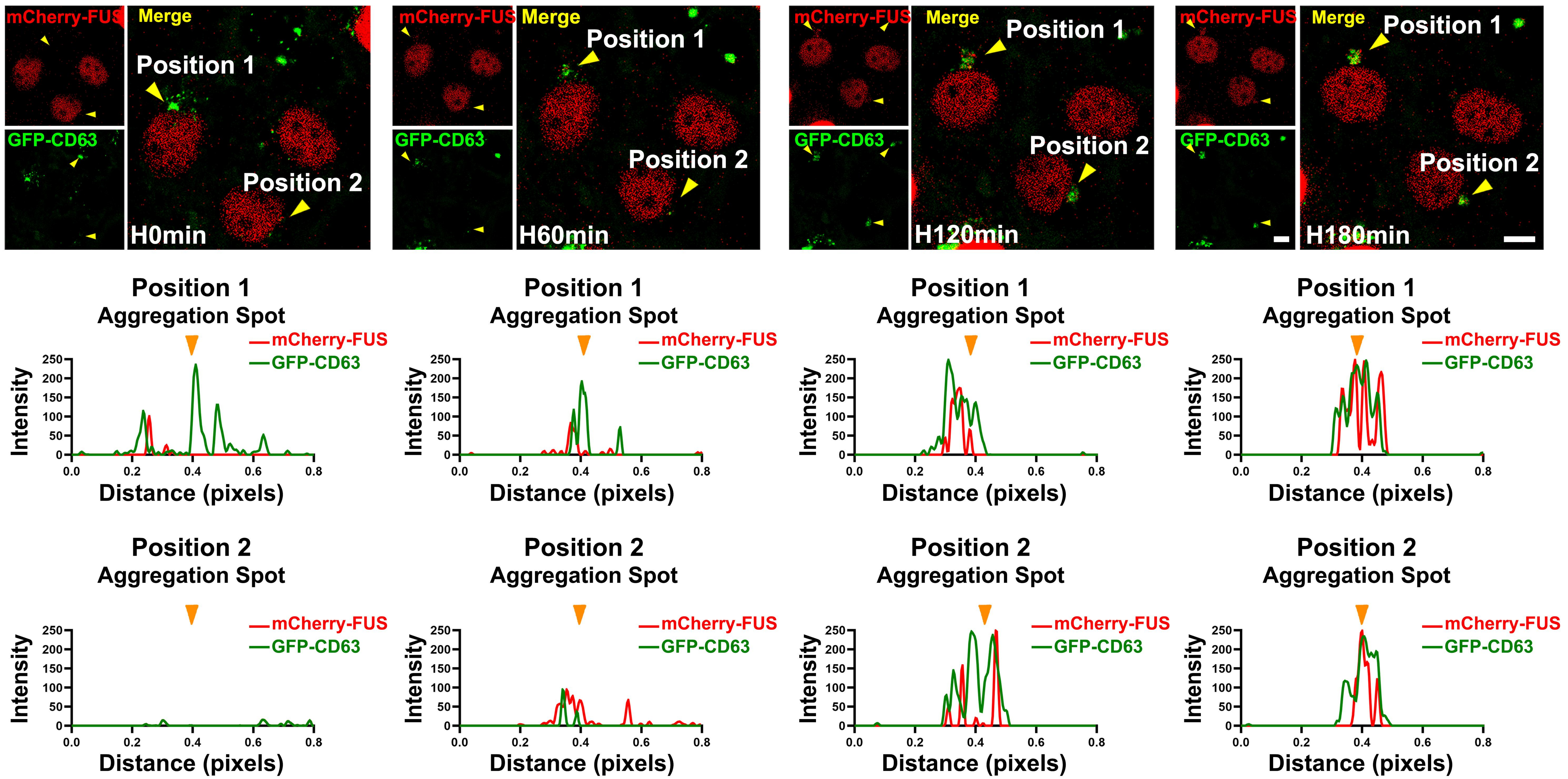

Figure S1. Time-lapse movie taken at 60-min intervals during the 180 min-hypoxia process.

Yellow arrows indicate long-lasting localization of GFP-CD63 and mCherry-FUS in transfected SH-SY5Y cells. H0min, H60min, H120min, and H180min indicate cells stressed under hypoxia for 0, 60, 120, and 180 min, respectively. The fluorescence intensity quantification of signals in each staining region is shown in the bottom diagrams. The overlap of different channels indicates co-localization between molecules. Scale bar, 20 μm.

Figure S2

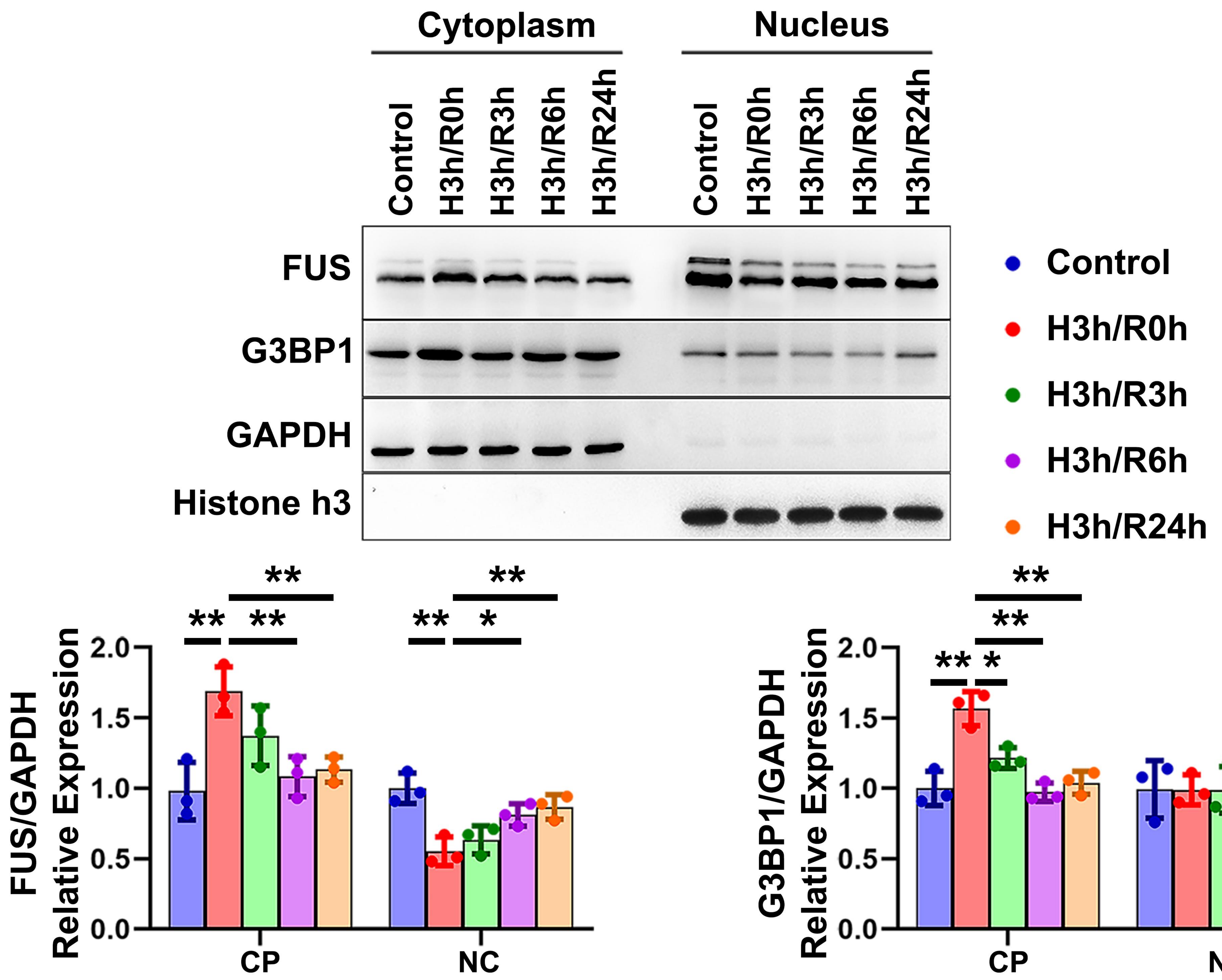

Fig S2. Detection of FUS and G3BP1 abundance in cytoplasm and nuclear from hypoxic cells

FUS and SG marker protein G3BP1 expressions in the cytoplasm (CP) and nuclear (NC) lysates from cells subjected to hypoxia and reperfusion at different time points (n=3 per group). GAPDH and Histone h3 were used as the loading controls for the cytoplasmic and nuclear fractions, respectively. *$p<0.05$; **$p<0.01$.

Figure S3

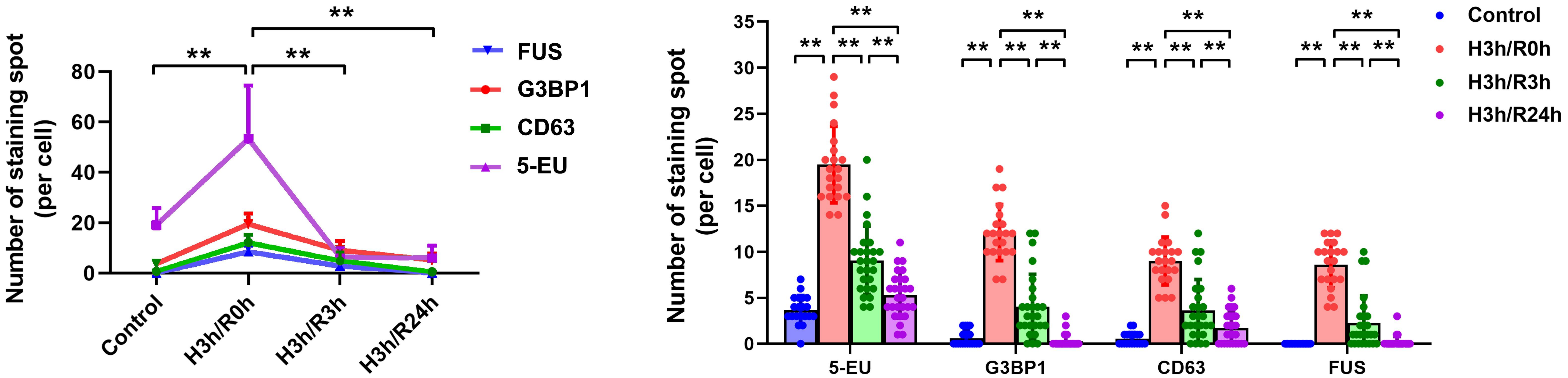

Figure S3. Quantification of the staining spots labeled by FUS, G3BP1, CD63 and the nonspecific RNAs in Figure 2A .

Nonspecific RNAs were labeled by 5-EU Click chemistry. n=9 cells from three independent experiments for measurement. **p<0.01.

Figure S4

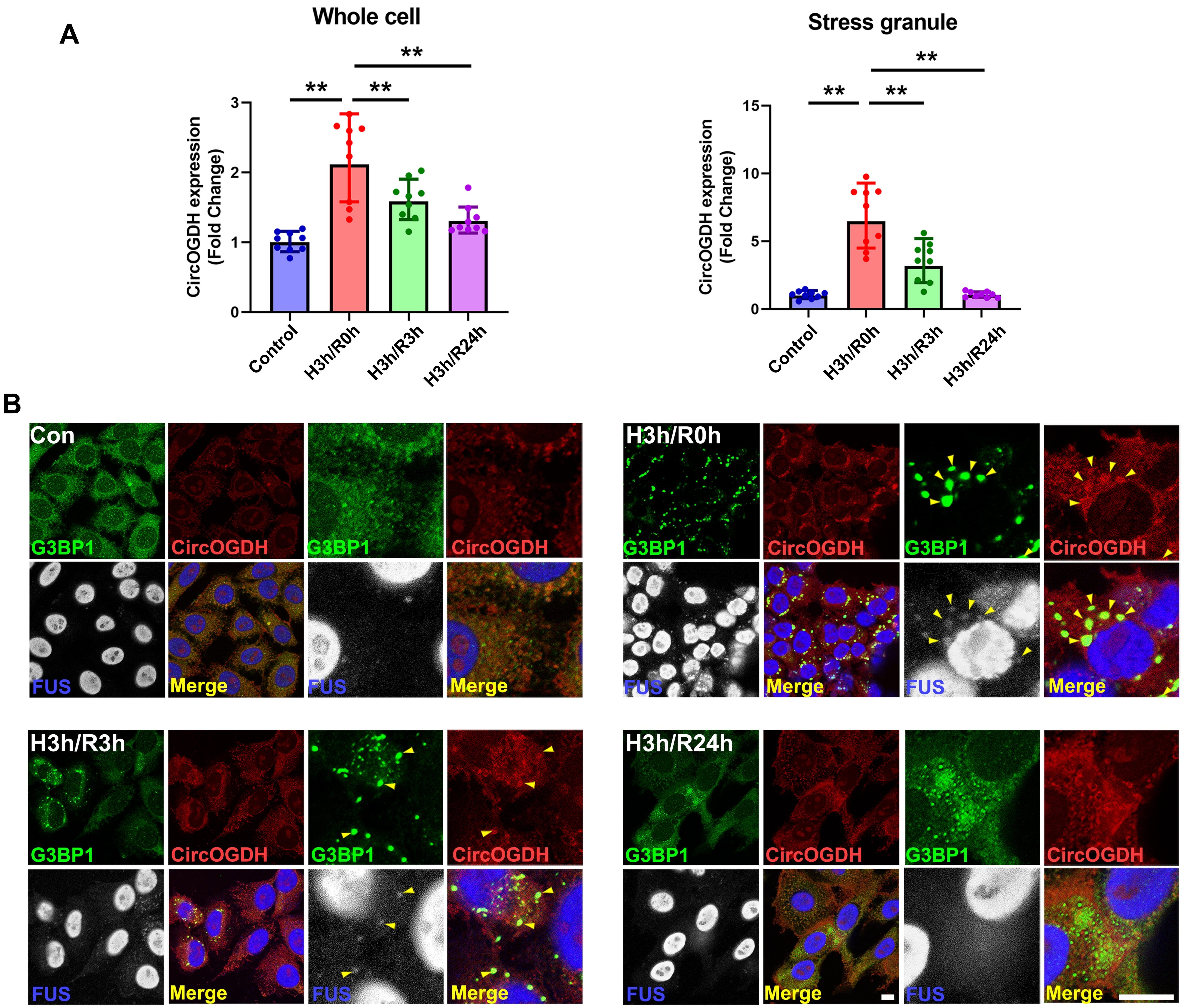

Figure S4. CircOGDH and FUS aggregate within SGs during hypoxia, and dissipate as SGs disassemble upon the relief of stress.

A. QPCR detection of CircOGDH in whole cell and SGs samples from Control and hypoxia cells with or without repersusion. Quantification of CircOGDH Ct values obtained from three biological replicates with three technical replicates in whole cell samples, and two technical replicates in SGs sample. 18S was used to normalize Ct values. **p<0.01.

B. Co-location staining of FUS with SG marker protein G3BP1 and CircOGDH in different cells, corresponding to Figure S4a.Yellow arrows indicate the co-staining points of G3BP1, FUS and CircOGDH. Scale bar, 20 μm.

Figure S5

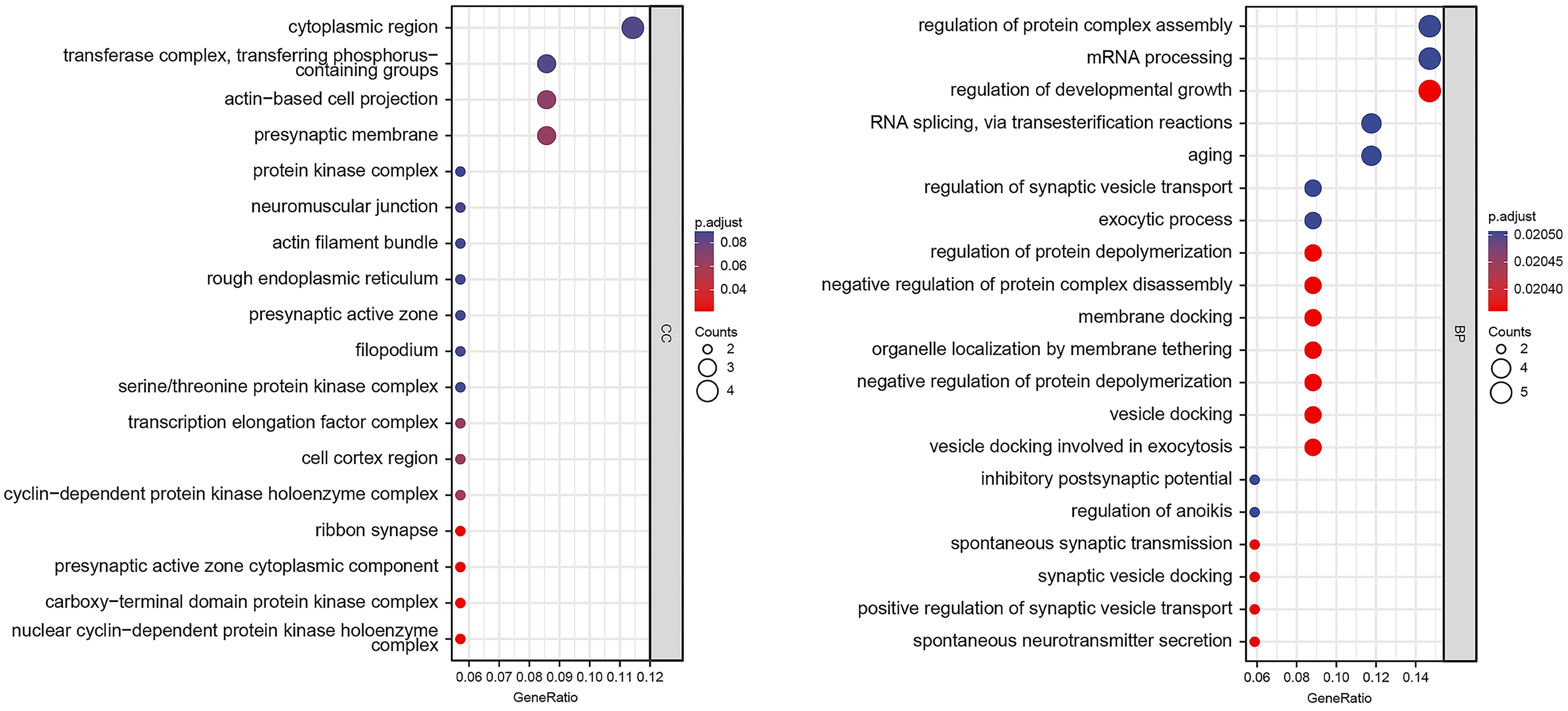

Fig S5. Biological Process and Cell Components enrichment analysis of nascent differential expressed circRNAs in hypoxic and control neuronal sEVs.

Figure S6

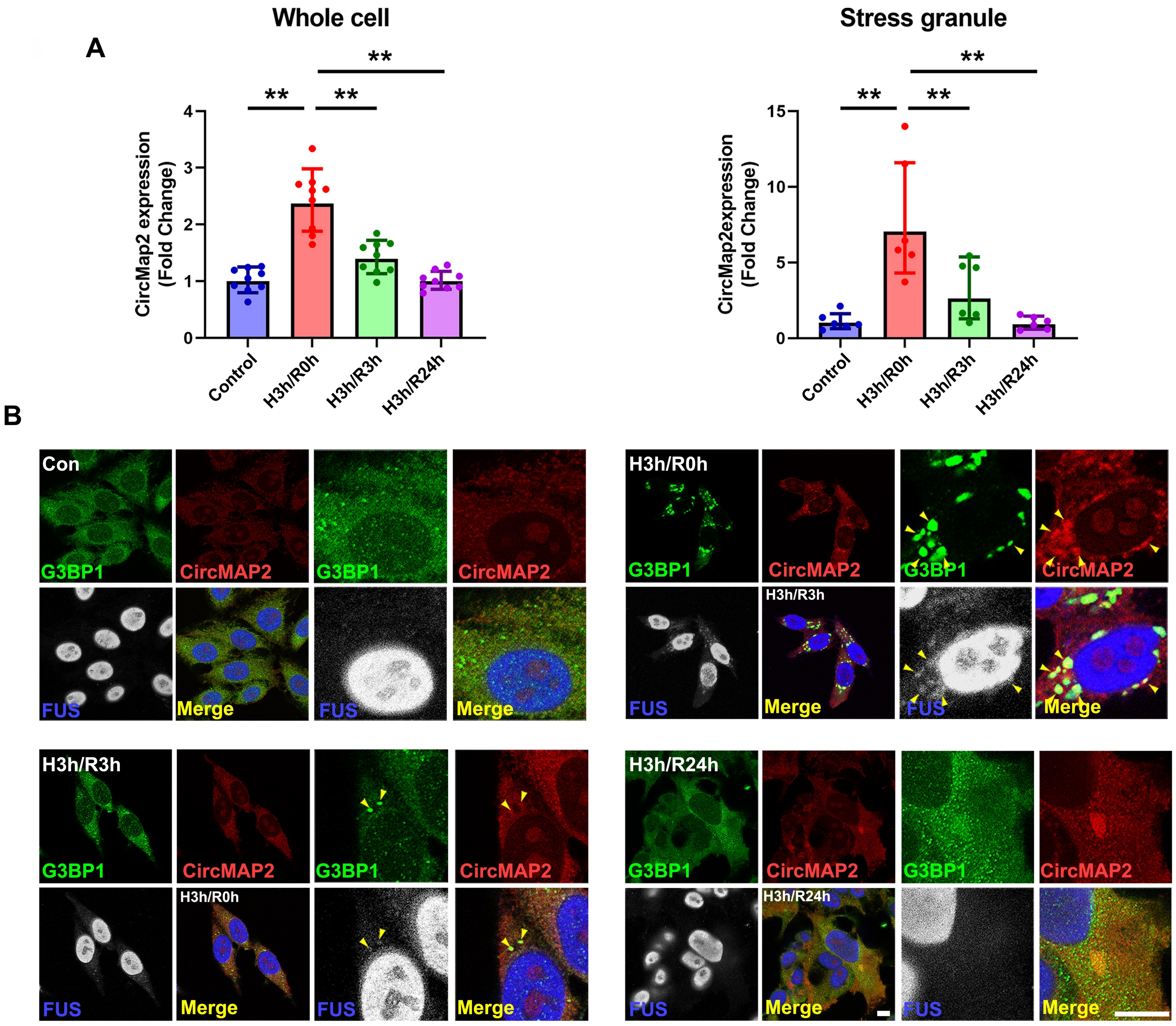

Figure S6. CircMAP2 and FUS aggregate within SGs during hypoxia, and dissipate as SGs disassemble upon the relief of stress.

A. QPCR detection of CircMAP2 in whole cell and SGs samples from Control and hypoxia cells with or without reperfusion. Quantification of CircMAP2 Ct values obtained from three biological replicates with three technical replicates in whole cell samples, and two technical replicates in SGs sample. 18S was used to normalize Ct values. **p<0.01.

B. Co-location staining of FUS with SG marker protein G3BP1 and CircMAP2 in different cells, corresponding to Figure S6a.Yellow arrows indicate the co-staining points of G3BP1, FUS and CircMAP2. Scale bar, 20 μm.

Figure S7

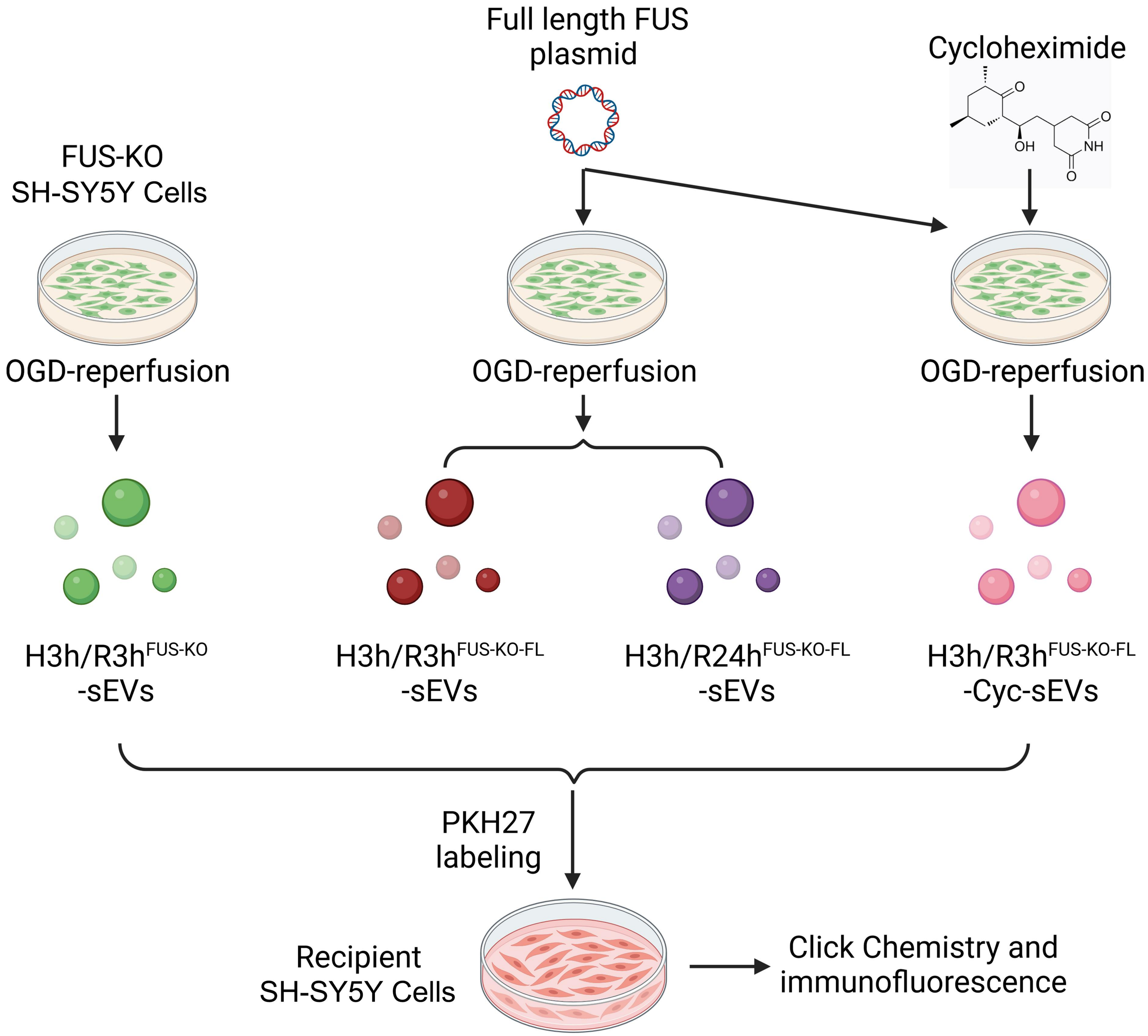

Figure S7. The workflow diagram for the cell treatment and sEVs grouping in Figure 3J.

KO represents FUS knockout; FL represents full length FUS; Cyc represents 200 μg/ml Cycloheximide treatment; H3h/R3h represents cells subjected to 3 hours of hypoxia followed by 3 hours of reperfusion; H3h/R24h represents cells subjected to 3 hours of hypoxia followed by 24 hours of reperfusion.

Figure S8

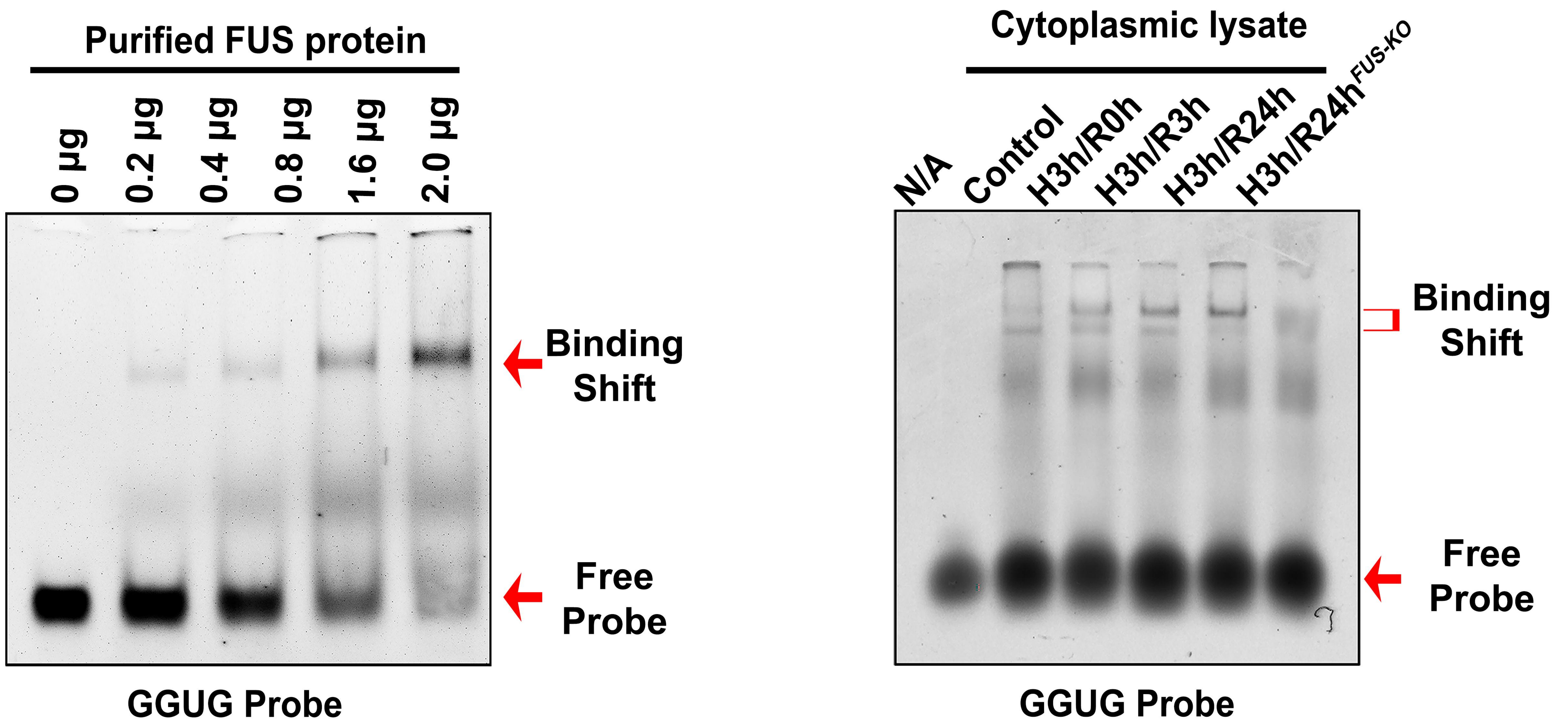

Figure S8. EMSA verification of the specific interaction between the GGUG probe and FUS.

GGUG probe with purified FUS peptides at different concentrations (left lane) and cytoplasmic lysates (right lane) from different treatment cells.

Figure S9

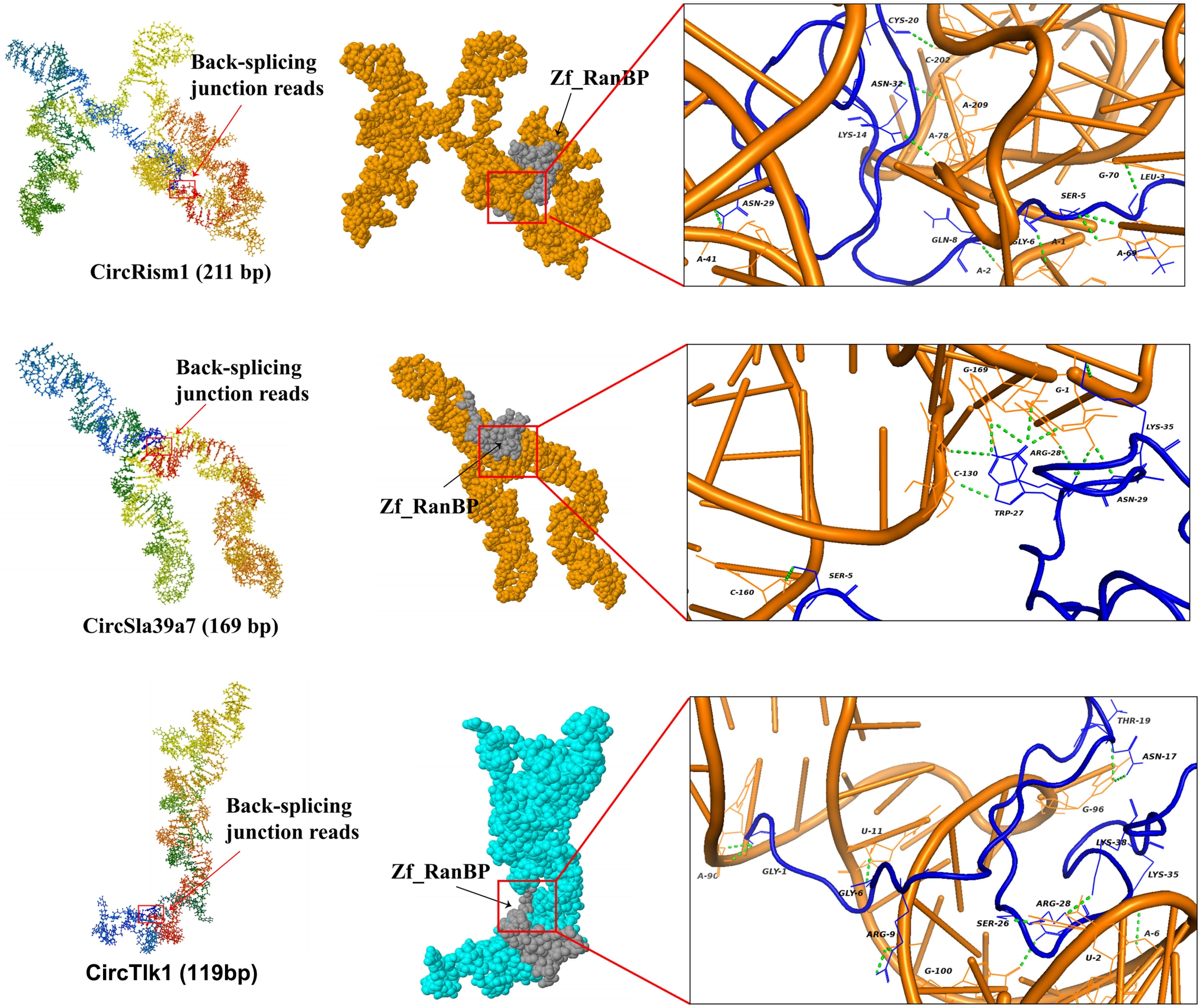

Figure S9 . Zf_RanBP domain of FUS selectively interacts with hypoxia related circRNAs by binding to their junction reads.

Prediction complex structures simulating Zf_RanBP domain combined with CircRism1, CircSla39a7 and CircTlk1. The left lane shows the predicted structures of circRNAs. In the middle lane, the complex structures of Zf_RanBP domain interacting with circRNAs are displayed. The right lane provides high-resolution views of the interaction regions between Zf_RanBP and the junction reads of circRNAs. The green dotted lines represent the covalent hydrogen bonds formed between the two molecules.

Figure S10

A

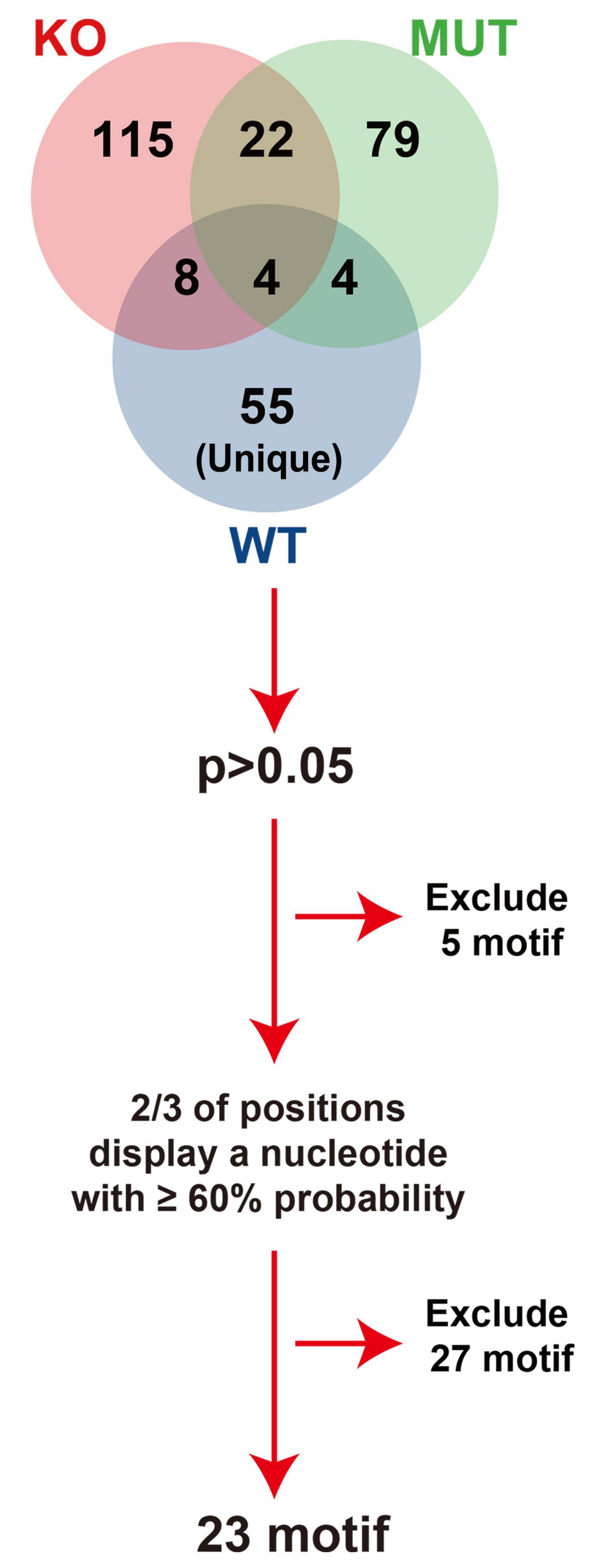

B

	WT unique motif					
	Motif	p value	Targets (%)	Background (%)	FDR	Fold Enrichment
4nt	CGAC	1.00E-21	46.07	28.94	0.002	1.59
	CGUU	1.00E-13	51.12	36.7	0.002	1.39
	CGAA	1.00E-13	26.23	17.78	0.002	1.48
	GCGU	1.00E-13	61.76	47.39	0.002	1.3
5nt	AUCGC	1.00E-16	52.61	36.83	0.002	1.43
	GGCUU	1.00E-06	40.63	31.78	0.002	1.28
	ACCGG	1.00E-05	10.16	5.62	0.002154	1.81
6nt	CGCUUA	1.00E-32	19.85	6.15	0.002	3.23
	UGCGUG	1.00E-20	17.35	6.69	0.002	2.59
	CGCCAA	1.00E-20	9.87	2.5	0.002	3.95
	CGCAUA	1.00E-18	4.01	0.4	0.002	10.02
	CUUUCG	1.00E-14	7.79	2.17	0.002	3.59
	CCGUAA	1.00E-12	11.03	4.3	0.002	2.57
	CGAAUC	1.00E-12	8.68	2.88	0.002	3.01
	CACUGA	1.00E-11	27.21	16.45	0.002	1.65
	CUAAAC	1.00E-11	37.26	25.23	a	1.48
	ACCGGA	1.00E-10	8.24	2.95	0.002	2.79
	AUACCG	1.00E-09	16.02	8.77	0.002	1.83
	UGGCUU	1.00E-09	29.04	19.21	0.002	1.51
	UAUACU	1.00E-09	12.59	6.08	0.002	2.07
	ACCCAA	1.00E-08	27.61	18.8	0.002	1.47
	GCGAUU	1.00E-07	7.44	3.11	0.002	2.39
	AAUAGG	1.00E-04	13.59	8.97	0.03808	1.52

Figure S10. Identification of FUS recognized RNAs motifs by eCLIP sequencing.

The hypoxic WT, FUS-KO and FUS-MUT cells were used for the eCLIP sequencing analysis. **A.** The Venn diagram and analysis flow chart of the WT cell specific motifs. **B.** The information of FUS-related motifs with high explicitness and statistically significant in A.
